# New nanostructure perovskite-based light-emitting diode with superior light extraction efficiency enhancement

**DOI:** 10.1038/s41598-024-55951-4

**Published:** 2024-03-06

**Authors:** Saeed Rahimi, Mehdi Eskandari, Davood Fathi

**Affiliations:** 1 Department of Electrical and Computer Engineering, Tarbiat Modares University (TMU), Tehran, Iran; 2 Nanomaterial Research Group, Academic Center for Education, Culture & Research (ACECR) on TMU, Tehran, Iran

**Keywords:** Optoelectronic devices and components, Inorganic LEDs, Nanophotonics and plasmonics

## Abstract

The external quantum efficiency (EQE) of a perovskite-based light-emitting diode (PELED) is a key indicator, comprising the internal quantum efficiency (IQE) and light extraction efficiency (LEE). Currently, enhancing EQE faces a major challenge in optimizing LEE. This study introduces an innovative structure to boost LEE, exploring various influencing parameters. The transition from a planar to a domical architecture leverages factors like the waveguiding effect, resulting in a remarkable tenfold increase in LEE, from 6 to 59%. Additionally, investigations into factors affecting LEE, such as altering dipole orientation, material-substrate contact angle, and layer thickness, reveal the potential for further improvement. The optimized structure attains an impressive LEE value of 74%.

## Introduction

In recent years, extensive research has been done regarding the use of perovskite materials in optoelectronic devices. The unique features of these materials have led researchers to further study their properties and applications. Their most obvious application is their use in the manufacturing of lasers^1–4^, light-emitting diodes^5–9^, and solar cells^10–16^. the prominent features of perovskites are long carrier penetration length (about one micron)^17,18^, easy and low-cost production^19^, production of purer light^20,21^, simplicity in the output light spectrum adjustment^22–25^, and etc. But considering all the mentioned advantages, perovskites also have disadvantages; The most important of which are structural instability^26–28^, the existence of many trap centers because of low trap formation energy^29–32^, low lifespan^33,34^ and low resistance to environmental factors such as humidity, oxygen, heat, and UV rays^35–37^.

The fabrication of the first PELED was reported in 1992 by Hong et al.^38^; however, due to the high loss, the temperature of PELED has increased greatly, and for this reason, the initial tests on this part have been carried out at a low temperature (77 Kelvin). Afterward, Era et al., in 1994, were able to produce a green PELED, which can produce light with a brightness of 10000 cd/m^2^ at a temperature of 77 Kelvin^39^. In 1996, the use of two-dimensional perovskite materials in the manufacturing of light-emitting diodes was reported by Hattori et al. and they were able to report an EQE value of 2.8% at a temperature as high as 110 Kelvin^40^. But the feasibility of these devices at low temperatures made the research in this field to be less developed. After many years, Koutselas et al. reported the construction of the first PELED with a color spectrum at room temperature in 2011, but they did not provide any information about its efficiency and operation^41^. Finally, since 2014, with the production of PELED at room temperature and using 3D perovskite materials by Smith et al., the beginning of serious activities in the research and development of these devices have been started^42–44^. Since then, the progress rate and the number of researches have increased day by day, as well as the significantly higher efficiency achievements for PELEDs by performing operations such as improving the process of making the perovskite layer, using nanostructures, improving the morphology of the layers, changing the architecture of the structure, photon recycling, using better carrier transfer layers, and etc.^29,30,33^. So far, the presented reports indicate that the efficiency of PELEDs has improved significantly and reached values higher than 20%^8,37,45,46^, and even in some visible colors it can be up to 50%^47^.

The efficiency of a PELED is calculated using Eq. (1), which essentially represents the ratio of the number of emitted photons from the device, to the total number of electrons injected into the device.

1 $${\text{EQE}} = {\text{IQE}} \times {\text{LEE}}$$

2 $${\text{IQE}} = {\text{B}}_{{{\text{e}} - {\text{h}}}} \times {\text{L}}_{{{\text{e}} - {\text{h}}}} \times {\text{PLQY}}$$

where EQE represents the output quantum efficiency, which can be used to compare the overall performance of PELEDs, and is obtained from the product of two efficiencies: IQE and LEE. IQE is considered to be the device’s ability to receive carriers and convert them into photons. It can also be referred to as an electrical-to-optical conversion efficiency. LEE, on the other hand, can be regarded as the ability of the structure to efficiently transfer generated light out of the device, and can be recognized as a purely optical efficiency. LEE is also known as the light extraction efficiency, which refers to the ratio of emitted light to the total generated light in the structure, and acts as a factor between the two aforementioned equations.

In Eq. (2), factors affecting the value of IQE are expressed, which show the ratio of the generated photons inside the structure to the injected electrons. IQE depends on three factors: the balance between injected carriers (B_e-h_), the conversion rate of electron–hole pairs to photons (PLQY), and the proportion of parasitic losses of electrons and holes (L_e–h_)^42^. In recent years, the PLQY parameter has increased significantly and has even reached a value near one^48,49^; however, LEE remained very low, behaving as the most restrictive efficiency parameter in PELEDs. Studies have shown that various factors contribute to the reduction of LEE, including the total internal reflection (TIR) phenomenon, losses and absorption in different layers, surface plasmonics, and base material losses^49–53^.

Among all the factors affecting η_out_ in PELED, the most important and effective factor is the TIR phenomenon, which is caused by the difference in refractive indices of different layers. Usually, the refractive indices of perovskite materials are higher than 2^42^. adjacent layers have a high refractive index difference compared to perovskite, which increases the TIR phenomenon. Increasing the TIR results in trapping more photons inside the perovskite layer, leading to reduced efficiency, increased temperature, and decreased lifetime of the perovskite. In addition to TIR, other factors such as the absorption rate of constituent materials, losses in the substrate material, and occurrence of surface plasmon polariton (SPP) phenomenon at the boundary of silver and ETL layers are also effective in reducing light extraction efficiency.

In this article, we aim to overcome the most important factors reducing LEE by using Finite Difference Time Domain (FDTD) simulation method and simulation of a new structure and showing the effect of structure architecture on the amount of light output. Also, the effect of each of the main factors in reducing light output will be measured and the changes in these factors will be examined in two planar and domical structures. The domical structure will be used to change the performance of the TIR phenomenon, as the most important factor for reducing LEE into a factor for increasing LEE. By changing the structural architecture from planar to domical, the amount of TIR will decrease and will be added to the amount of LEE. In addition to examining the main parameters affecting LEE, the effect of factors such as the orientation angle of dipole oscillations, the contact angle of material with the surface, active layer thickness, electron transfer layer thickness, and hole transfer layer thickness will be evaluated. By optimizing some of these structural factors, the best conditions for increasing light output from the introduced structure will be achieved.

The structure presented in the article is a new and innovative structure. The main difference between this structure and other structures presented in the past that have used patterned surfaces is that in this structure, all the layers forming the piece are made in the form of hemisphere shell. And finally, the entire PELED structure is placed in a silver coating, producing a PELED cell with a radius of less than one micron. These PELED cells are completely separate from each other, which allows us to use the waveguiding phenomenon as a positive factor in increasing LEE. Also, it should be noted that due to the presence of separate cells in the design of the structure, there will be a high degree of flexibility in the designed structure. The presented structure is a constructible structure and an attempt has been made to use available and common layers that have been used in previous reports^54–56^. Glass serves as a transparent layer and the substrate for the piece, and ITO is used as an electrode due to its transparency and excellent carrier transfer ability. PEDOT: PSS and F8 materials have also been used in the construction of numerous organic, polymer, and perovskite LEDs as electron and hole transporting layers, and their performance has been studied. Perovskite material is MAPbBr3 that commonly used in green-colored PELEDs, and its emission spectrum is in the green color range. Additionally, silver is known as one of the most popular metals in the construction of electronic devices and their electrodes.

### Simulation method

**Figure 1:**
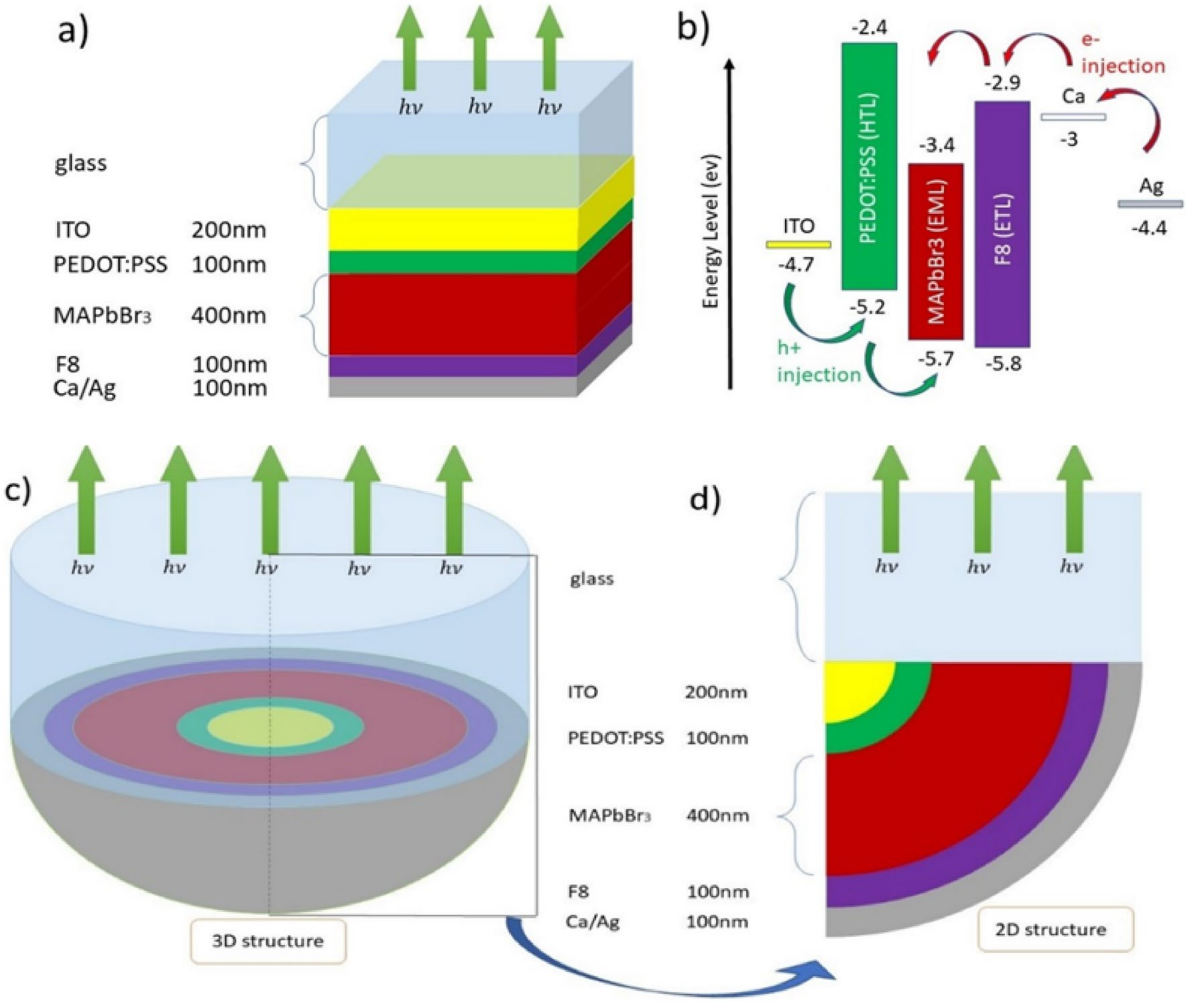
Overview of the studied structures: (**a**) planar structure, (**b**) Energy band diagram related to the materials used in PELED, (**c**) 3D view of the domical structure, and (**d**) 2D view of a section of the domical structure and its constituent layers.

At the beginning of this study, a planar PELED structure was used as a reference for comparing light output. The desired structure is composed of 5 different layers: ITO layer with a thickness of 200 nm as the anode electrode, PEDOT: PSS layer with a thickness of 100 nm as the hole transport layer (HTL), MAPbBr3 layer with a thickness of 400 nm as the active or light-emitting layer (EML), F8 layer with a thickness of 100 nm as the electron transport layer (ETL) and silver (Ag) layer with a thickness of 100 nm as the cathode electrode. The schematic image of the planar PELED structure is shown in Fig. 1a.

In addition, the energy band diagram of this structure is illustrated in Fig. 1b. All materials were selected based on previous studies to ensure proper electrical transfer, stress between layers, and performance of these materials when combined with each other^54,55^. Furthermore, sources for the extraction of parameters related to the refractive index and loss coefficient of various materials are mentioned in Table 1.

**Table 1:** Material details for the PELED structure.

Material name	Abbreviation	Function	Thickness (nm)	References
Indium tin oxide	ITO	Anode	200	^59^
Poly(3,4-ethylenedioxythiophene): poly(styrenesulfonate)	PEDOT: PSS	Hole transport layer (HTL)	100	^60^
Methylammonium lead tribromide (CH3NH3PbBr3)	MAPbBr3	Emission layer (EML)	400	^61^
Poly(9,9′-dioctyl-fluorene)	F8	Electron transport layer (ETL)	100	^62^
Calcium/silver	Ca/Ag	Cathode	100	^63^

In the simulation process, the FDTD computational method has been utilized to solve the Maxwell's equations inside the structures. The calculation method with the help of FDTD is explained in detail in the Supplementary section and its equations are also mentioned. Only the optical behavior of the structure has been evaluated, and the effect of the structure's architecture on the generated photons has been investigated to assess the light extraction efficiency (LEE) parameter. In a PELED, all generated light is produced from electron–hole radiative recombination and is emitted in all directions; hence, the generated light can be modeled as a dipole oscillation. For this purpose, a point-like light source that produces light in a dipole oscillation manner has been used, and the generated light by the point light source is emitted in all directions.

Due to Purcell's phenomenon or the spontaneous emission of light in resonators, the amount of generated light by the dipole oscillation differs from the injected-light value into the structure^57,58^. To correct this issue, a small box made of measurement monitors has been used to determine the actual injected light value. Additionally, several other monitors have been employed to measure and investigate the output light intensity.

In order to improve the light output, a second structure according to Fig. 1c has been proposed, which has the same materials and thicknesses as the planar structure, with the only difference being the domed shape of the second structure. In this structure, the constituent materials of the PELED are arranged in a hemispherical shape inside each other, with the innermost layer being ITO and the outermost layer being Ag. Figure 1d also shows a two-dimensional image of the domed structure. In this structure, an increase in the light output has been attempted by using the reflection effect of the silver layer and its curved shape.

## Results and discussion

The light generated in the active layer of a PELED is emitted in all directions. Some of it, which hit the common boundary between layers at a 90-degree angle, will pass through the boundary and enter the next layer. However, as the angle of incidence deviates further from the perpendicular angle, more light will be refracted during its passage through the boundary, leading to total internal reflection (TIR) and trapping of the light within the structure. This phenomenon inside the structure of a PELED is called wave guiding mode, and it leads to reduced light output and efficiency while increasing the temperature of the structure. Unfortunately, perovskite materials have weak ionic bonds, and low resistance to environmental factors such as heat, moisture, oxygen, UV waves, and etc. In addition, their decomposition rate increases significantly with an increase in the structure's temperature. Therefore, by improving the light extraction efficiency of the structure, one can also extend the lifetime of a PELED.

**Figure 2:**
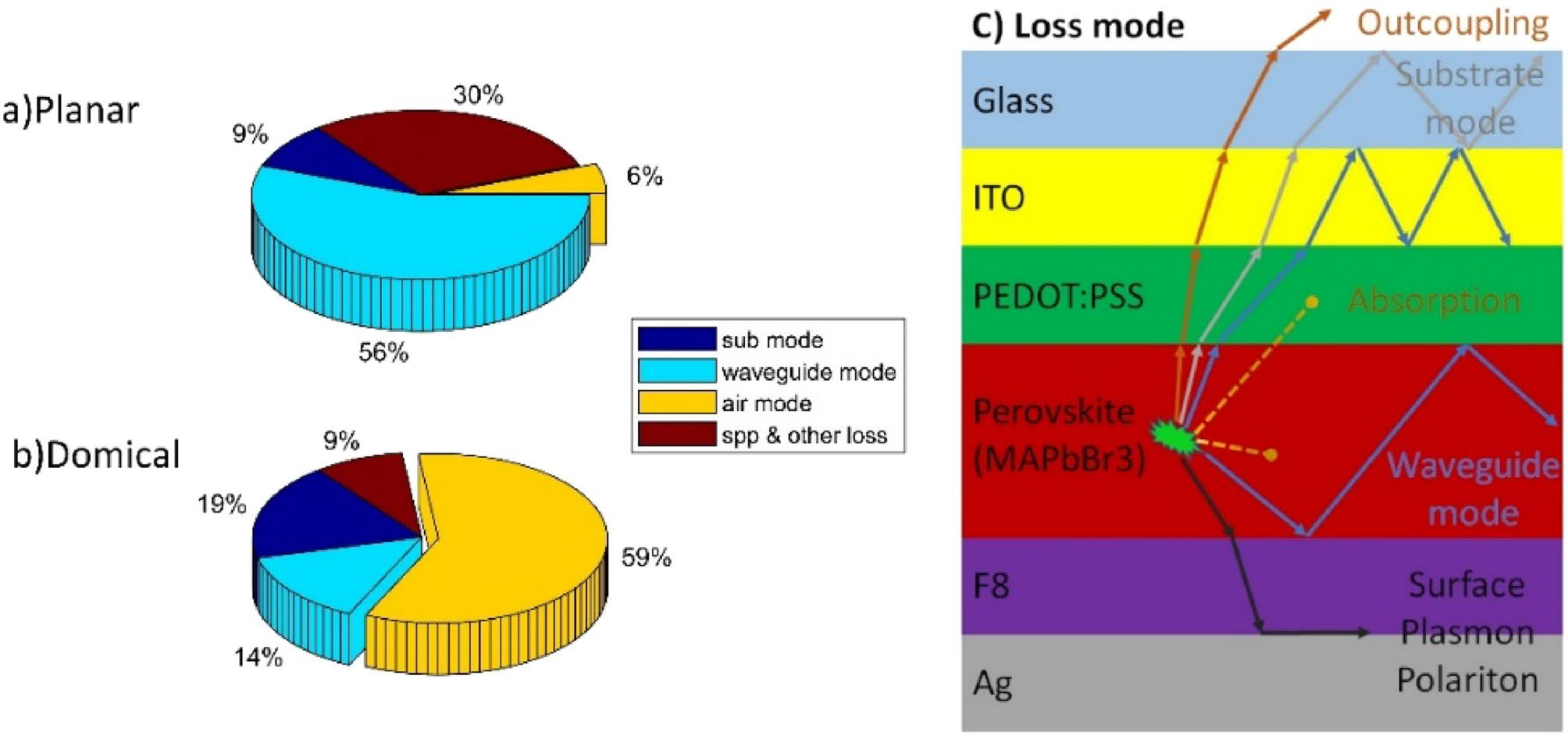
Amount of losses and light output in (**a**) Planar structure, (**b**) Domical structure, and (**c**) Representation of different types of losses in a planar PELED structure that is divided into 4 different modes; (1) SPP or Surface Plasmon Polariton generated only at the interface of the silver metal layer and F8. (2) Waveguide mode, which is resulted from total internal reflection inside the structure due to the difference in the refractive indices of various layers and can occur in all layers but is only applicable to the trapped light rays in Perovskite, ETL, HTL, and ITO layers. (3) Absorption, which appears due to the absorption property of various materials and can be observed in all layers. (4) Substrate mode, which occurs due to the trapped light rays inside the glass substrate layer and is completely similar to the waveguide mode, but due to the high thickness of the glass layer, it is evaluated as a separate factor in the losses.

In order to investigate the light extraction efficiency, all factors affecting this parameter must be evaluated. Generally, four factors can be considered in reducing the effective coefficient. These factors include the total internal reflection (TIR) phenomenon, which occurs due to the difference in the refractive indices of various layers, absorption of different materials, which depends on the type of materials used in the structure, the surface plasmonic effect (SPP), which occurs due to the presence of a metallic layer in the structure, and losses due to the substrate material. According to the conducted research, the most important factor in reducing light extraction is the TIR phenomenon. This phenomenon occurs at all common boundaries between layers. Figure 2 illustrates the amount of light output from the structure along with the amount of losses for each of the above-mentioned factors, in two planar and domical structures. In Fig. 2a, the effect of the TIR phenomenon can be clearly seen, which traps 56% of the total radiative power inside the structure, creates a large portion of losses, and can increase the temperature of the structure and reduce the lifespan of PELEDs. The amount of light output from the planar structure is about 6%, which is a small value and indicates the inefficiency of planar structures. A similar graph for the domical structure is shown in Fig. 2b, where the amount of light output is approximately 59%, confirming more than 9.83 times the increase in light output from the structure and a significant reduction in waveguide losses. Figure 2c also shows a clear display of the types of losses in a simple and planar PELED structure.

**Figure 3:**
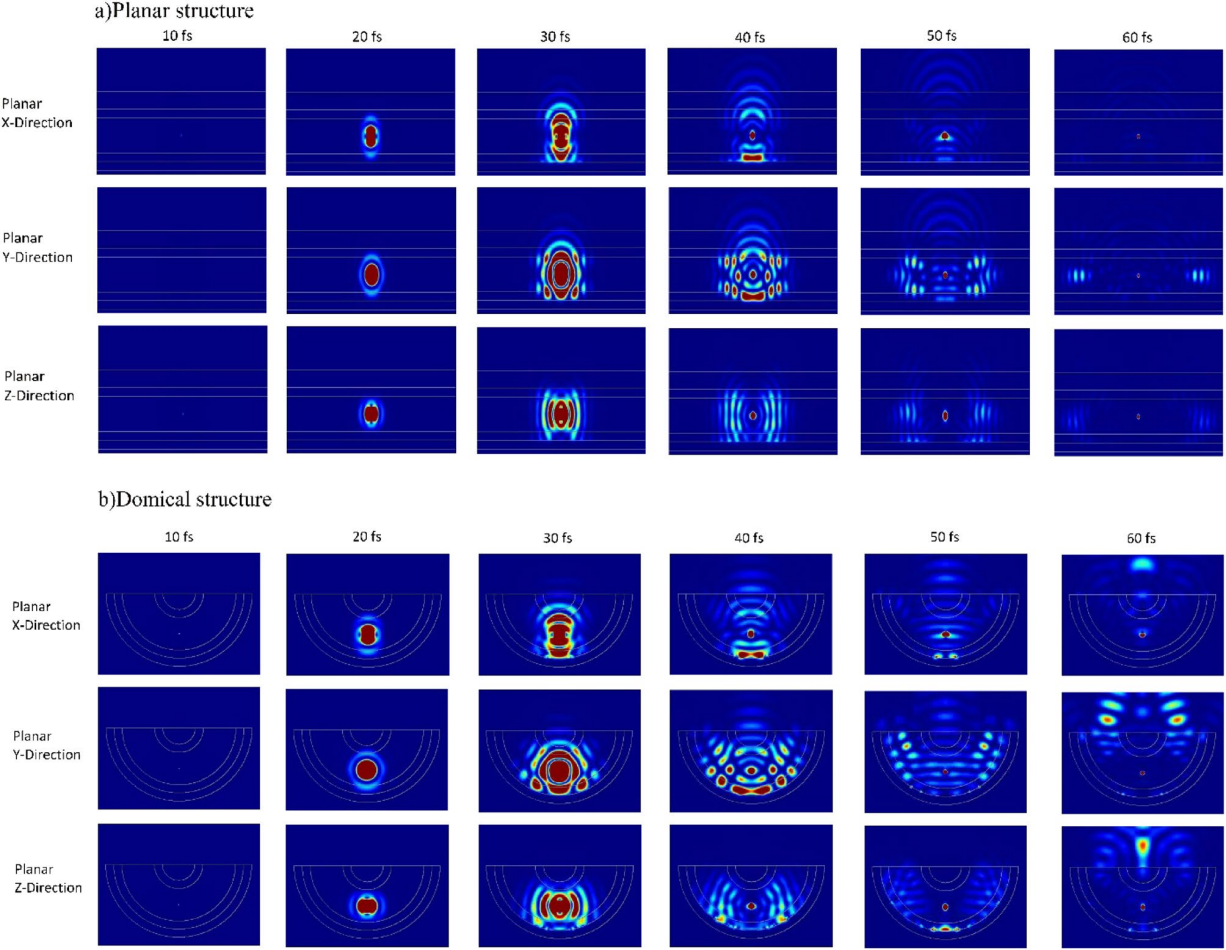
The electric field profile in two structures: (**a**) planar and (**b**) domical. In both structures, the electric field profile for three different dipole orientations along the X, Y, and Z directions is shown at six different times. In both field profiles, the phenomenon of light trapping is clearly evident; however, in the planar structure, this phenomenon causes light to be trapped inside the perovskite channel, increasing structural losses. In contrast, in the domical structure, due to the connection of the endpoints of the perovskite channel to the glass substrate, most of the light exits. The complete video of the electric field profile in both structures is provided in the Supplementary section.

The difference in behavior between the two structures presented above can be easily understood by examining Fig. 3a and b. In Fig. 3a, the field profile for different times is shown for a planar structure. As it can be seen, the emitted light inside the perovskite layer after colliding with the metal layer underneath the structure and also the substrate layer on top of the structure resulted in intense reflection. This phenomenon caused the light to return inside the structure, and the planar structure to behave like a waveguide, transferring most of the emitted light horizontally, which is not desirable and leads to an increase in losses and inadequate light output. However, in Fig. 3b, which corresponds to the field profile in a domical structure, the ease of light output from the structure can be observed. After generating light in the perovskite layer, the emitted light is dispersed in all directions, the same as the planar structure, which resulted in internal reflection and is transferred along the perovskite layer. However, as can be seen, part of the perovskite layer is connected to the substrate layer, and the majority of the output light enters to substrate layer from that area and then it is transferred outside the structure. Essentially, it can be said that the performance of the domical structure is like a curved waveguide, and the light generated at the center of this waveguide is extracted from the structure using its waveguide property, which improves the amount of output light from the structure. In fact, in the domical structure, the TIR phenomenon has been used to increase the amount of output light. Domical structures convert most of the optical power that is lost in the planar structure due to the waveguiding into the optical power that exits. In simpler terms, the performance of the domical structure is like a bent waveguide, and the light generated at the center of this waveguide is exited from the structure with the help of its waveguiding properties, which improves the amount of the output light from the structure. By carefully examining Fig. 2, more points can be deduced. The amount of waveguide phenomenon inside the domical structure has decreased significantly compared to the planar structure. Showing only a value close to 14%, which can be attributed to the difference in refractive indices between the perovskite channel and the glass substrate and also light leakage from the perovskite channel to adjacent layers, which causes light to be trapped in the layers. By comparing Fig. 2a and b, a decrease in the losses related to SPP & other losses can also be observed, which can be attributed to the ease of light exiting the domical structure and not getting trapped inside the structure. In the planar structure, the light is confined inside the layers, especially the perovskite layer, and due to its inability to exit, it causes multiple reflections. Under these conditions, the probability of light colliding with the surface of the silver layer and the probability of SPP will increase. The trapped light, due to its inability to exit, will be absorbed through the different layers, ultimately causing an increase in the aforementioned losses inside the planar structure. However, the only negative point in Fig. 2 is the increase in the amount of substrate layer losses, which includes a small amount of total power and can be attributed to more light transfer to this layer; the values presented in Fig. 2 indicate the available power for each mode relative to the total power. It is quite obvious that increasing the input power to the substrate layer can increase this layer’s losses relative to the total generated power.

One of the important and investigable points during simulation is the orientation of dipole oscillation. In all simulations, the dipoles' orientation is omnidirectional, i.e., three dipoles in the X, Y, and Z directions are used. It should be noted that the orientation in the X and Y directions is referred to as horizontal directionality, while the orientation in the Z direction is called vertical directionality. In the simulations performed, the effects of each orientation of dipole oscillations have been assumed to be equal. This means that a total power radiation value of 1/3 has been considered for each direction of coordinate axes. Considering that the orientations in the X and Y directions are similar to each other in both discussed structures and represent the horizontal directionality, the total radiation power in the structure can be expressed by Eq. (3), where α is equal to 2/3 and represents the effect of horizontal orientation on the total power, P_horizontal_ indicates the light power in the horizontal orientation and P_vertical_ indicates the light power in the vertical orientation^64^.

3 $${\text{P}}_{{{\text{Total}}}} = \, \alpha {\text{P}}_{{{\text{Horizontal}}}} + \left( {{1} - \alpha } \right){\text{P}}_{{{\text{Vertical}}}}$$

**Figure 4:**
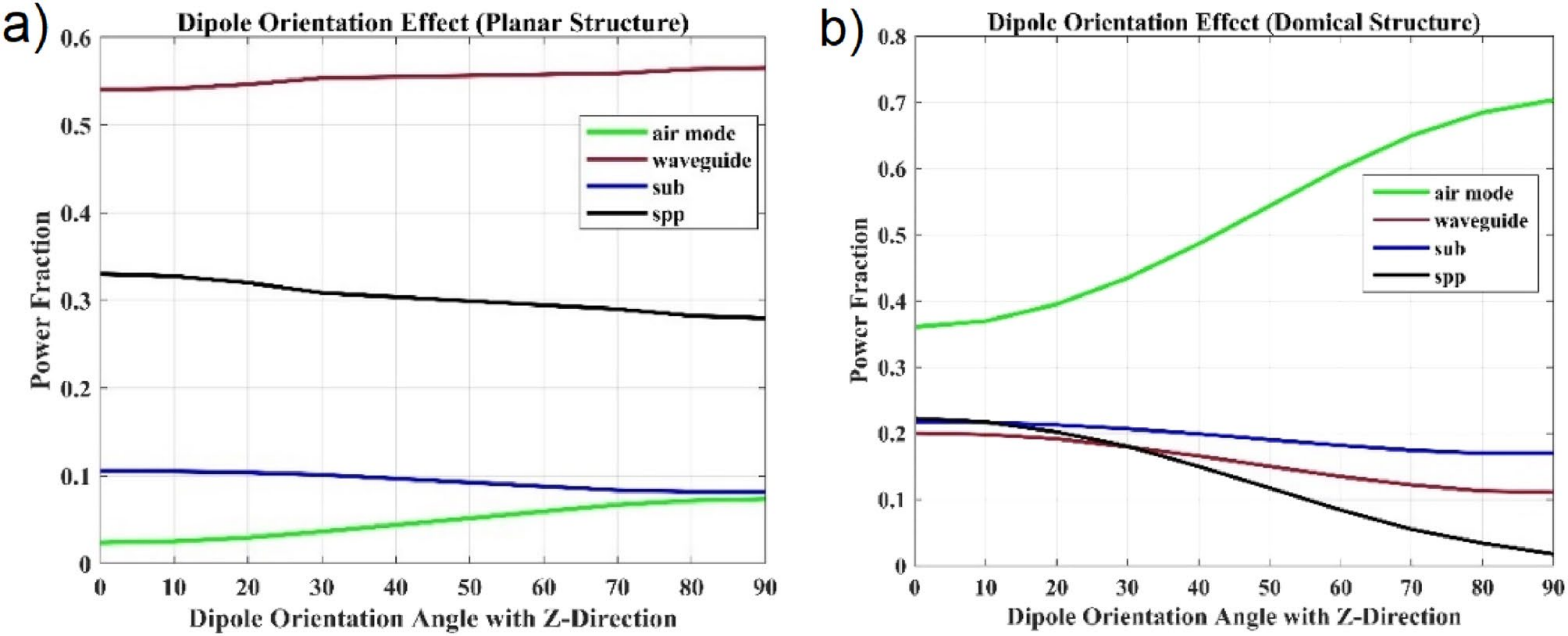
The amount of optical power for output light and loss factors, according to the dipole orientation, in two structures: (**a**) planar and (**b**) domical. In the above graphs, the 0-degree angle on the horizontal axis indicates the orientation along the Z-axis, and the 90-degree angle represents the orientation along the X or Y-axis.

Several simulations have been performed to investigate the effect of dipole orientation on the output light from both structures, in which the dipole orientation angle changes from the vertical state (Z direction) to the horizontal state (X direction) with 10-degree steps. The simulations indicate that in a planar structure, the effect of dipole orientation on the output light is greater than that in a domical structure. As shown in Fig. 4a, the output light from the planar structure (green curve) is equal to 2.38% in the vertical direction, which is the minimum amount of output light. However, the output light increases with the change in dipole orientation angle and reaches 7.35% in the horizontal direction, showing a significant increase of 3.09 times in the output light. Similar simulations have been performed for the domical structure, and its results are shown in Fig. 4b. In this structure, the amount of output light in the vertical direction is equal to 36.04%, and in the horizontal direction, it increases by 1.95 times to 70.33%. This decrease in the effect of dipole orientation on the amount of output light is attributed to the curved shape of the domical structure. In a planar structure, the overall surface of the structure is uniform, and the light will be the same for all particles or points on these surfaces with different directions. However, in the domical structure, the dipole orientation is only completely perpendicular or horizontal for a small part of the structure, and for other points, the orientation of dipole is accompanied by an angle. Therefore, the dipole orientation will not be absolute for all points in the domical structure and it will always be a combination of all three directions. Consequently, the susceptibility of output light in the domical structure will be far less than that of the planar structure. This is a positive factor in increasing the amount of output light from the structure since the light will be scattered at all angles and has the probability of any direction. Plus, the less the impact of the dipole orientation, the more light will be exited from the structure.

In Fig. 4a, the amount of emitted light changes with the variation of the dipole orientation. As the dipole orientation approaches the horizontal position, the amount of emitted light increases. This increase is proportional to the reduction of loss factors such as SPP and sub. In Fig. 4a, the black curve represents the amount of losses in the SPP state. The SPP phenomenon only occurs in TM mode, in a planar structure, this mode can be considered to be oriented in the Z and Y direction, and the TE mode can be considered to be oriented in the X direction^65,66^. Therefore, as the dipole orientation moves from the Z axis towards the X axis, the amount of SPP losses decreases because we are essentially moving from the TM polarization to the TE polarization. The decrease in SPP losses means that less light power is absorbed at the interface between the silver layers and the ETL layer. Therefore, the amount of reflection from the metallic layer increases, which enhances the waveguiding phenomenon. In other words, since the waveguiding phenomenon is proportional to the amount of light reflection from adjacent layers, increasing the reflection from the metallic layer will enhance this factor, which is indicated by the red color curve in Fig. 4a. In Fig. 4b, the amount of output light has increased with the horizontal dipole orientation, which is mainly due to the reduction in SPP losses. In the domical structure, by creating curvature in the structure, an effort was made to use the waveguiding phenomenon as a positive factor in output light. Due to this curvature in structure, it is not possible to accurately consider TE and TM modes with dipole orientation in the X and Z directions. In fact, the polarization along the X axis will only exist for a small part of the structure as a pure TE mode, and for other points, it will be a combination of both modes. The notable point in Fig. 4b is that the amount of SPP has decreased significantly, which can be a factor in increasing waveguiding, but considering the architecture of the structure and the use of the waveguiding phenomenon to increase output light, the decrease in SPP has shown itself as an increase in output light.

**Figure 5:**
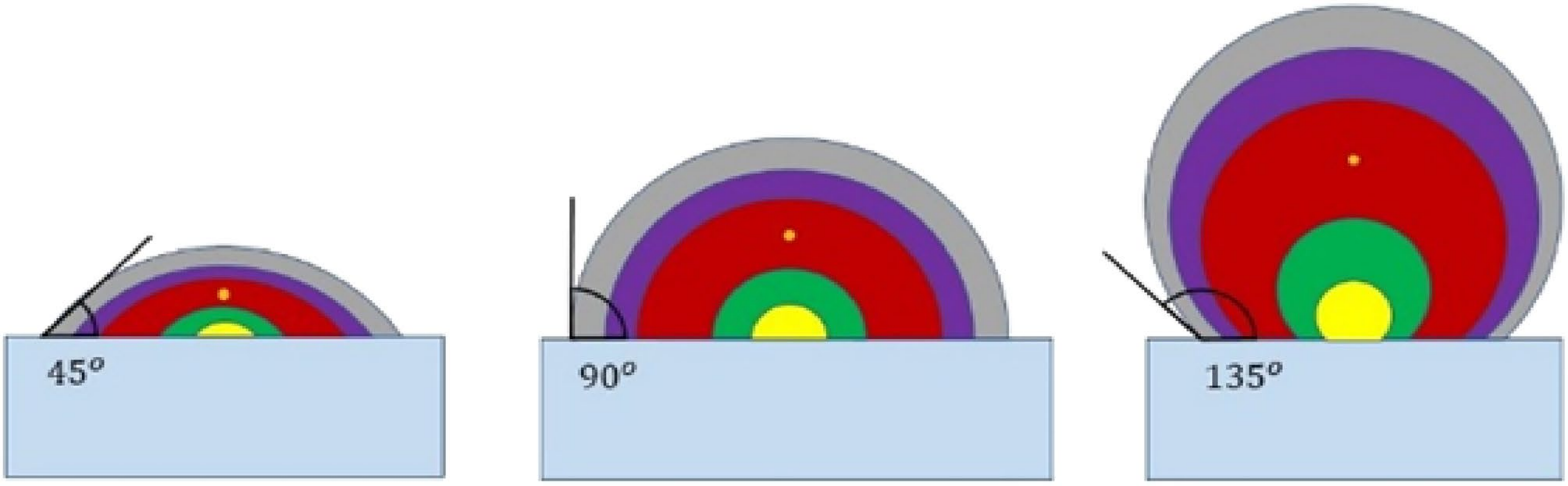
Illustration of several contact angles with a glass substrate. From left to right, the contact angles are 45, 90, and 135°. In the above images, the volume of all materials should be constant, therefore, the radii of the half spheres were taken into account and their centers were also changed accordingly.

Another aspect evaluated in this article is the contact angle of different materials on the glass substrate. All simulations were performed for a domical structure assuming a 90-degree contact angle between different materials and the substrate. Here, by examining different contact angles between materials and the substrate layer, the best possible conditions for increasing light output have been sought. This action has been taken for two reasons. First, to optimize the structure and find the best value for the contact angle of materials and the substrate layer to maximize output light. Secondly, considering that most of the deposition layers in PELED are formed using materials in the liquid phase, the material in question can have different contact angles depending on environmental conditions and deposition parameters. On the other hand, the term contact angle is used for fluids, but in the PELED structure, the materials will ultimately be in the solid phase. Even if the contact angle between materials and the surface can be simulated in the liquid phase, it cannot be guaranteed that it will not change after being converted to the solid phase^67^. Therefore, it is necessary to investigate different contact angles. Based on the explanations provided above, it can be concluded that measuring the contact angle of materials with different surfaces can only be performed in the laboratory and after the manufacturing or coating process. It is not possible to accurately predict and simulate the contact angle, therefore, different contact angles have been considered for Perovskite and other materials, as shown in Fig. 5, to evaluate various conditions for this structure. The contact angle between materials and the substrate layer has been examined from 30° to 150° with 5-degree increments. An important point in simulating the domical structure with different contact angles is the constant volume of materials, especially the active material. Therefore, changing the contact angle will also change the centrality and radius of the hemispheres present in the structure, which is reflected as an increase or decrease in thickness at various points of the structure, according to Fig. 5. The equations and calculation methods for the values related to the radius and location of the center of the hemispheres are mentioned in the Supplementary section according to Supplementary Fig. S1, and the corresponding table for these values is presented in Supplementary Tables S1 and S2. The volume of material in the structure is also mentioned in Supplementary Table S3.

**Figure 6:**
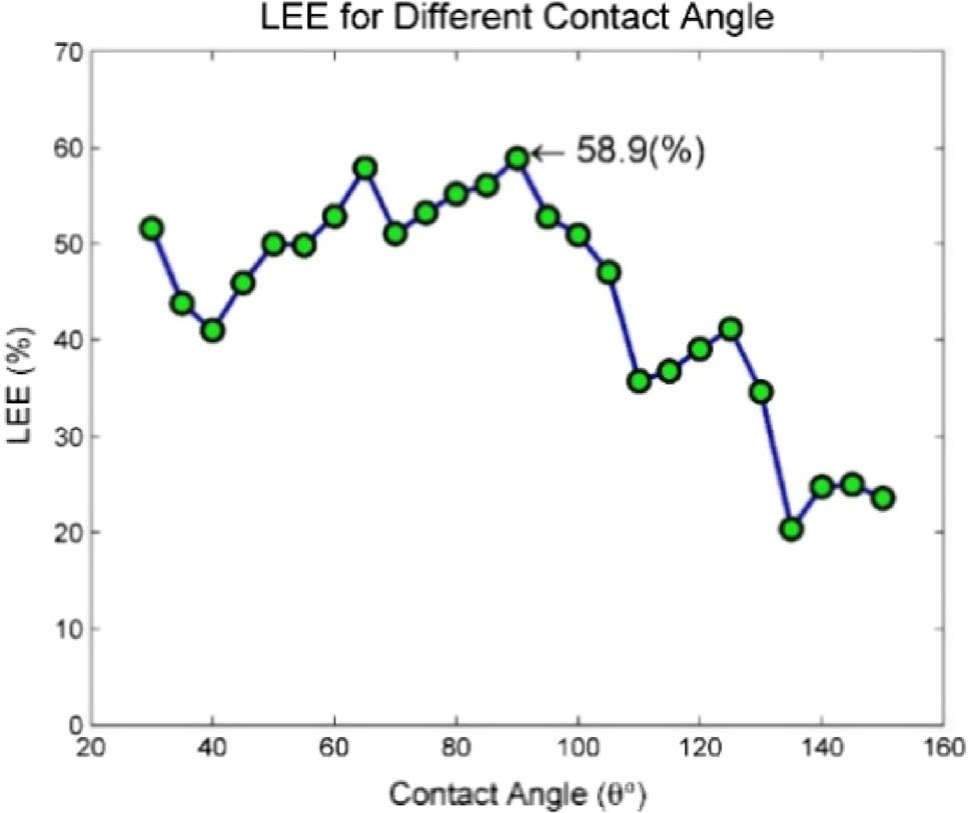
The amount of output light from the structure depending on the different contact angles between the materials forming the structure and the substrate layer.

The results of the simulation for different contact angles are shown in Fig. 6, indicating the highest amount of output light near the 90-degree angle. As mentioned above, predicting the actual contact angle between different materials is not possible; however, it is important to note that the contact angle between materials depends on various factors such as the temperature of the coating, environmental humidity, material type, surface roughness, etc., which can significantly change this angle by altering them^68–70^.

The Supplementary Figs. S2–S8 displays the images of the field profile at contact angles of 30°, 50°, 70°, 90°, 110°, 130°, and 150° for a better understanding of Fig. 6. Based on the mentioned figures, the behavior of the Fig. 6 can be justified. When the contact angle is close to 30°, the transmitted light through the perovskite channel undergoes severe reflection, while passing through the boundary between perovskite layer and the substrate material. As a result, they reflect into the structure and after colliding with ETL and silver layers and reducing their power, they leave the structure. This phenomenon leads to a decrease in the output light and an increase in losses. The same phenomenon occurs when the incident angle is close to 150°. The reason behind the severe reflection of light during its exit from the perovskite channel is that the boundary between the perovskite channel and the substrate material is at a steep angle at these contact angles. Moreover, the light waves inside the channel are largely parallel with waveguide channel due to the multiple internal reflections on the channel walls, which causes the incident light to collide with the boundary at an angle greater than the critical angle and undergo total internal reflection.

The obtained results in Fig. 6 confirm that the maximum amount of light output coefficient is achieved at a 90° angle. Therefore, to achieve the highest efficiency, we should use factors that affect the contact angle to bring it closer to 90°. Surface roughness is one of the physical factors affecting the contact angle and can also have a positive effect on the amount of output light. Numerous reports exist regarding the positive effects of surface roughness on increasing the amount of output light in various structures, and most of them justify this issue with the presence of different angles on the surface^51,71–73^. As shown in Supplementary Fig. S9, when roughness is created on the surface of the substrate material, the produced light in the center of PELED has a higher chance of hitting the surface at an appropriate angle. As demonstrated by the multiple beams in Supplementary Fig. S9, the interaction and output light from the structure in both rough and non-rough conditions are displayed. As it was seen, in completely planar structures, only rays that encounter the interface between the two layers at an angle less than the critical angle (approximately 40°) can be transmitted from the perovskite layer to the glass layer. Rays with larger angles than critical angle are subjected to reflection and return inside the structure. However, in cases where the interface between the two layers is rough, rays with angles greater than the critical angle can pass through the interface and transmit to the substrate layer. This is due to the fact that the surface between the two layers has different angles, and when light rays collide with these surfaces, the probability of hitting an appropriate angle increases. This phenomenon can result in an increase in the amount of output light from the perovskite layer.

**Figure 7:**
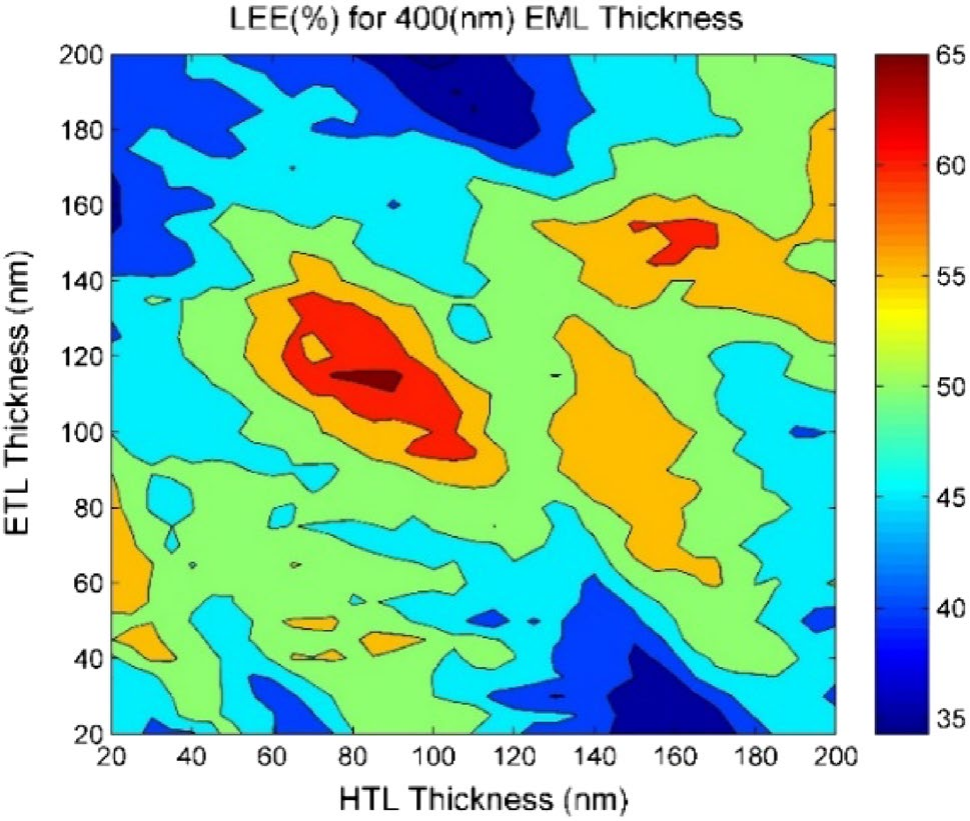
The amount of output light from the domical structure, with varying thicknesses of electron transport layer (ETL) and hole transport layer (HTL) for the perovskite layer with a fixed thickness of 400 nm at the wavelength of 545 nm.

One of the effective factors on the amount of output light is the thickness of the constituent layers of a structure, such as perovskite material and carrier transport layers. As shown in Fig. 1, both evaluated structures have different layers, and these layers can act as an amplifier, enhancing or attenuating the intensity of light at certain wavelengths. These wavelengths are dependent on the thicknesses of the various layers and the differences in their refractive indices. Given the constant wavelength, the thickness of the layers must be determined to enhance the generated wavelength in the perovskite material and avoid attenuation. Based on simulation results, it can be concluded that changing the thickness of electron and hole transport layers will have a considerable effect on output light. In Fig. 7, the value of the light extraction efficiency is shown for perovskite with a thickness of 400 nm and different thicknesses of electron and hole transport layers, between 20 to 200 nm, with steps of 5 nm. The best values are achieved at a thickness of 90 nm for the HTL layer and 115 nm for the ETL layer, where the efficiency is close to 66.34%. The thickness of the perovskite layer also affects the amount of output light. Therefore, images S10 to S27 show the amount of output light for different thicknesses of the perovskite layer, from 50 to 950 nm with 50-nm steps, in the Supplementary section. In these simulations, the thicknesses of the electron and hole transport layers have been varied by 20 nm steps. By considering the presented images and performing various simulations, the optimal thickness for achieving the highest output light coefficient can be determined. The highest value of 73.77% is achieved for a perovskite layer with thickness of 50 nm, an electron transport layer with thickness of 160 nm, and a hole transport layer with thickness of 140 nm. The results for the highest amount of output light for the presented shapes in the Supplementary section and in Table S4 are provided.

In conclusion, it can be stated that the domical structure presented in this article possesses prominent optical features, which justify its construction. To build this structure, attention must be paid to key points, including establishing a connection between the ITO domes and anode contact, the method for fabricating dome-shaped layers, utilizing low-temperature deposition techniques, and so on. The proposed construction method for this structure is thoroughly explained in the Supplementary section.

## Conclusion

In this article, we attempted to calculate the LEE for two planar and domical structures using the FDTD method and evaluate the factors affecting it. The value of LEE was calculated to be 6% in the planar structure, and the loss factors that reduced LEE were also investigated. Among them, the TIR phenomenon was identified as the main loss factor with a value of 56%. Due to the high losses of the TIR phenomenon, efforts have been made for the first time to utilize a new structure (dome structure) to turn this negative factor into a positive factor and increase light output. The distinguishing feature of the research presented in this report is that, in most of the articles and reports previously published, efforts have been made to increase productivity by eliminating or reducing the detrimental effects of certain factors. However, due to the inherent nature of some of these detrimental factors, the work done has often not had a significant impact. In this article, instead of simply trying to overcome the detrimental factor, attempts were made to improve the amount of light output by changing the type of influence this factor has. The amount of output light was increased to nearly 59%, which indicated 10 times increase. In the next step, to optimize the structure, some factors affecting the LEE, such as the thickness of the electron and hole transport layers, the thickness of the perovskite layer, and the contact angle of the materials on the substrate layer were evaluated. For the perovskite layer with a thickness of 400 nm, the optimal thicknesses of ETL and HTL were calculated to be 115 and 85 nm, respectively. Also, the highest amount of output light was determined for a contact angle of 90 degrees. However, the optimal structures for different thicknesses of the perovskite layer were also identified in the Supplementary section, with the highest value of LEE obtained for a perovskite layer with the thickness of 50 nm being 73.77%. The results of this study clearly demonstrate the extent to which changing the architecture of the structure can be effective in increasing the level of output light. Moreover, it can be said that the output light from the side edges of the planar PELED, which is considered as the waveguiding loss, can be converted into a factor for increasing output light and increasing the overall efficiency of the system. The simulation results in the planar structure are highly corresponding to the laboratory results, and implementing this method on a domical structure will provide reliable results that can be used in analyzing and optimizing other structures.

### Supplementary Information

Supplementary Information.

## Data Availability

The data that support the findings of this study are available from the corresponding author upon reasonable request.
