# Supplementary material for: New nanostructure perovskite-based light-emitting diode with superior light extraction efficiency enhancement
Source: Sci Rep. 2024 Mar 6;14:5500. doi: 10.1038/s41598-024-55951-4 (PMC10918065; doi:10.1038/s41598-024-55951-4)
Supplement: Supplementary file 1 — Supplementary Information. [file 41598_2024_55951_MOESM1_ESM.docx]

**Supplementary section**

**FDTD method**

To analyze the behavior of electromagnetic waves in a specific structure, the Maxwell's equations need to be solved for that structure. There are various methods available for this purpose, one of which is the Finite-Difference Time-Domain (FDTD) method. In this method, the Maxwell's equations are solved for a specific time period. The equations used in this method are as follows:

$\nabla\times\vec{E}\left( t \right)=-\frac{\partial\vec{B}(t)}{\partial t}$ Eq.(S1)

$\nabla\times\vec{H}\left( t \right)=\frac{\partial\vec{D}(t)}{\partial t}$ Eq.(S2)

$\vec{B}\left( t \right)=\mu.\vec{H}(t)$ Eq.(S3)

$\vec{D}\left( t \right)=\varepsilon.\vec{E}(t)$ Eq.(S4)

Here, E represents the electric field, H represents the magnetic field, ε is the electric permittivity, μ is the magnetic permeability, B represents the magnetic flux density, and D represents the electric flux density. The two curl equations mentioned above are computed using the finite-difference method and are well-known as the finite-difference equations in the time domain due to their temporal derivatives. To solve these equations for a structure, the entire structure needs to be mesh in order to perform the differentiation operations. The mesh used in different structures in this article is chosen based on the dimensions of the layers and the significance of different regions, which ranges from 0.1 to 20 nanometers in various structures.

$\nabla\times\vec{E}\left( t \right)=-\mu\frac{\partial\vec{H}\left( t \right)}{\partial t} \Longrightarrow\nabla\times\vec{E}\left( t \right)\cong-\mu\frac{\vec{H}\left( t+\Delta t \right)-\vec{H}(t)}{\Delta t}$ Eq.(S5)

$\nabla\times\vec{H}\left( t \right)=\varepsilon\frac{\partial\vec{E}\left( t \right)}{\partial t} \Longrightarrow\nabla\times\vec{H}\left( t \right)\cong\varepsilon\frac{\vec{E}\left( t+\Delta t \right)-\vec{E}(t)}{\Delta t}$ Eq.(S6)

The time derivative is employed for differentiation, and its equations are given below:

$\nabla\times\vec{E}\left( t \right)=-\mu\frac{\partial\vec{H}\left( t \right)}{\partial t} \Longrightarrow\nabla\times\vec{E}\left( t \right)\cong-\mu\frac{\vec{H}\left( t+\Delta t/2 \right)-\vec{H}(t-\Delta t/2)}{\Delta t}$ Eq.(S7)

$\nabla\times\vec{H}\left( t \right)=\varepsilon\frac{\partial\vec{E}\left( t \right)}{\partial t} \Longrightarrow\nabla\times\vec{H}\left( t+\Delta t/2 \right)\cong\varepsilon\frac{\vec{E}\left( t+\Delta t \right)-\vec{E}(t)}{\Delta t}$ Eq.(S8)

In the above equations, the Yee grid is used for differentiation. This type of grid is compatible with the definition of the curl equations of Maxwell's equations and corrects the numerical errors in differentiation. Finally, the following equations, derived from the aforementioned equations, are utilized to calculate the electric and magnetic fields. These equations are known as the update equations. With the knowledge of the field values at one point, the field values at the next point can be calculated.

$\left. \vec{H} \right|_{t+\frac{\Delta t}{2}}=\left. \vec{H} \right|_{t-\frac{\Delta t}{2}}-\frac{\Delta t}{\mu}\left( \nabla\times\left. \vec{E} \right|_{t} \right)$ Eq.(S9)

$\left. \vec{E} \right|_{t+\Delta t}=\left. \vec{E} \right|_{t}+\frac{\Delta t}{\varepsilon}\left( \nabla\times\left. \vec{H} \right|_{t+\frac{\Delta t}{2}} \right)$ Eq.(S10)

For planar structures, periodic boundary conditions are used in the X and Y directions, and the Perfectly Matched Layer (PML) boundary condition is employed along the Z direction. However, in domical structures, due to the enclosure of the entire structure by a silver layer, there is no need for periodic boundary conditions, and PML boundary conditions are used in all directions.

To simulate light generation inside the active layer, a point dipole source is used. The fields generated by this source are applied to the structure, and with the help of the above equations, the values of electric and magnetic fields can be calculated at different points in the structure. Additionally, the amount of energy emitted from the structure can be calculated based on these field values.

**Calculate spherical cap parameters for contact angle simulation**


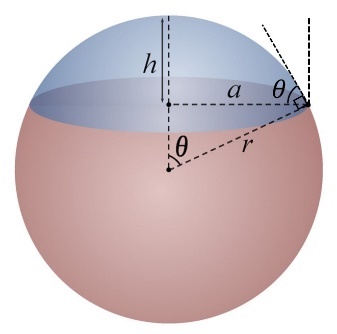


**Figure S1** shows how to calculate the volume of a spherical cap based on the contact angle and radius. The blue area represents the volume of the spherical cap, while the red area represents another part of the sphere that is not considered in the simulation.

To calculate the parameters related to spherical cap, which are essentially the layers that form the structure, Equation (S11) can be used:

$V=\frac{{\pi h}^{2}}{3}\left( 3r-h \right)$ Eq. (S11)

where parameter *V* represents the volume of each material, parameter *h* represents the height of the spherical cap, and *r* represents the radius of the sphere. In this equation, both *r* and *h* are unknown, and the contact angle parameter is also not included in the equation. Therefore, Equation (S12) is used to include the contact angle parameter in the calculation of the volume.

$h=r-rcos\theta$ Eq. (S12)

where the parameter θ represents the contact angle of the materials with the substrate layer. By combining the two equations, Equation (S11) and Equation (S12), the main equation for calculating the spherical cap parameters at each contact angle is obtained, as shown in Equation (S13):

$V=\frac{\pi r^{2}}{3}\left( 1-cos\theta\right)^{2}\left( 2+cos\theta\right)$ Eq. (S13)

By keeping the volume of the material constant and changing the contact angle, the values ​​of the sphere radiuses (r) are obtained, and by using the Equation (S12), the height of the spherical cap (h) can be calculated. Due to the fact that in the simulation process, the boundary of the substrate layer and the material is selected on the Z=-1μm plane, the position of the center of the sphere is calculated using the Equation (S14), where the reason for the presence of -1μm is this issue and the parameter Z represents the distance between the center of the sphere and the Z=0 plane.

$Z=rcos\theta-1\mu m$ Eq. (S14)

The results related to the values ​​of the radius and position of the sphere center from the boundary of the substrate layer are shown in Tables S1 and S2, respectively, and the values ​​of the constant volume of the material for all layers are presented in Table S3.

**Table S1** The radius of the hemisphere of the constituent materials of the structure at different contact angles.

| **Contact Angle** | **R_ITO (nm)** | **R-PEDOT: PSS (nm)** | **R-Perovskite (nm)** | **R-F8 (nm)** | **R-Ag (nm)** |
| --- | --- | --- | --- | --- | --- |
| **30˚** | 678 | 1020 | 237 0 | 2710 | 3050 |
| **35˚** | 558 | 837 | 1950 | 2230 | 2510 |
| **40˚** | 473 | 709 | 1650 | 1890 | 2130 |
| **45˚** | 410 | 615 | 1430 | 1640 | 1840 |
| **50˚** | 362 | 543 | 1270 | 1450 | 1630 |
| **55˚** | 325 | 487 | 1140 | 1300 | 1460 |
| **60˚** | 295 | 442 | 1030 | 1180 | 1330 |
| **65˚** | 271 | 406 | 947 | 1080 | 1220 |
| **70˚** | 251 | 376 | 878 | 1000 | 1130 |
| **75˚** | 234 | 352 | 821 | 938 | 1060 |
| **80˚** | 221 | 331 | 773 | 884 | 994 |
| **85˚** | 210 | 314 | 733 | 838 | 943 |
| **90˚** | 200 | 300 | 700 | 800 | 900 |
| **95˚** | 192 | 288 | 672 | 768 | 864 |
| **100˚** | 185 | 278 | 648 | 741 | 834 |
| **105˚** | 180 | 269 | 629 | 719 | 808 |
| **110˚** | 175 | 262 | 612 | 700 | 787 |
| **115˚** | 171 | 257 | 599 | 685 | 770 |
| **120˚** | 168 | 252 | 588 | 672 | 756 |
| **125˚** | 165 | 248 | 579 | 662 | 745 |
| **130˚** | 163 | 245 | 572 | 654 | 736 |
| **135˚** | 162 | 243 | 567 | 648 | 729 |
| **140˚** | 161 | 241 | 563 | 643 | 724 |
| **145˚** | 160 | 240 | 560 | 640 | 720 |
| **150˚** | 159 | 239 | 558 | 638 | 717 |

**Table S2** The center of the hemispheres of the constituent materials of the structure at different contact angles.

| **Contact Angle** | **Z_ITO (nm)** | **Z-PEDOT: PSS (nm)** | **Z-Perovskite (nm)** | **Z-F8 (nm)** | **Z-Ag (nm)** |
| --- | --- | --- | --- | --- | --- |
| **30˚** | -413 | -120 | 1050 | 1350 | 1640 |
| **35˚** | -543 | -315 | 599 | 828 | 1060 |
| **40˚** | -638 | -457 | 268 | 449 | 630 |
| **45˚** | -710 | -565 | 14.6 | 160 | 304 |
| **50˚** | -767 | -651 | -186 | -69.2 | 47.2 |
| **55˚** | -814 | -721 | -348 | -255 | -162 |
| **60˚** | -853 | -779 | -484 | -411 | -337 |
| **65˚** | -886 | -828 | -600 | -543 | -485 |
| **70˚** | -914 | -871 | -700 | -657 | -614 |
| **75˚** | -939 | -909 | -788 | -757 | -727 |
| **80˚** | -962 | -942 | -866 | -847 | -827 |
| **85˚** | -982 | -973 | -936 | -927 | -918 |
| **90˚** | -1000 | -1000 | -1000 | -1000 | -1000 |
| **95˚** | -1020 | -1030 | -1060 | -1070 | -1080 |
| **100˚** | -1030 | -1050 | -1110 | -1130 | -1140 |
| **105˚** | -1050 | -1070 | -1160 | -1190 | -1210 |
| **110˚** | -1060 | -1090 | -1210 | -1240 | -1270 |
| **115˚** | -1070 | -1110 | -1250 | -1290 | -1330 |
| **120˚** | -1080 | -1130 | -1290 | -1340 | -1380 |
| **125˚** | -1090 | -1140 | -1330 | -1380 | -1430 |
| **130˚** | -1110 | -1160 | -1370 | -1420 | -1470 |
| **135˚** | -1110 | -1170 | -1400 | -1460 | -1520 |
| **140˚** | -1120 | -1180 | -1430 | -1490 | -1550 |
| **145˚** | -1130 | -1200 | -1460 | -1520 | -1590 |
| **150˚** | -1140 | -1210 | -1480 | -1550 | -1620 |

**Table S3** The volume of materials used in the structure (m^3^)

| **V_ITO** | **V_PEDOT: PSS** | **V_Perovskite** | **V_F8** | **V_Ag** |
| --- | --- | --- | --- | --- |
| 1.6755E-23 | 3.9793E-23 | 6.6183E-22 | 3.5395E-22 | 4.5448E-22 |


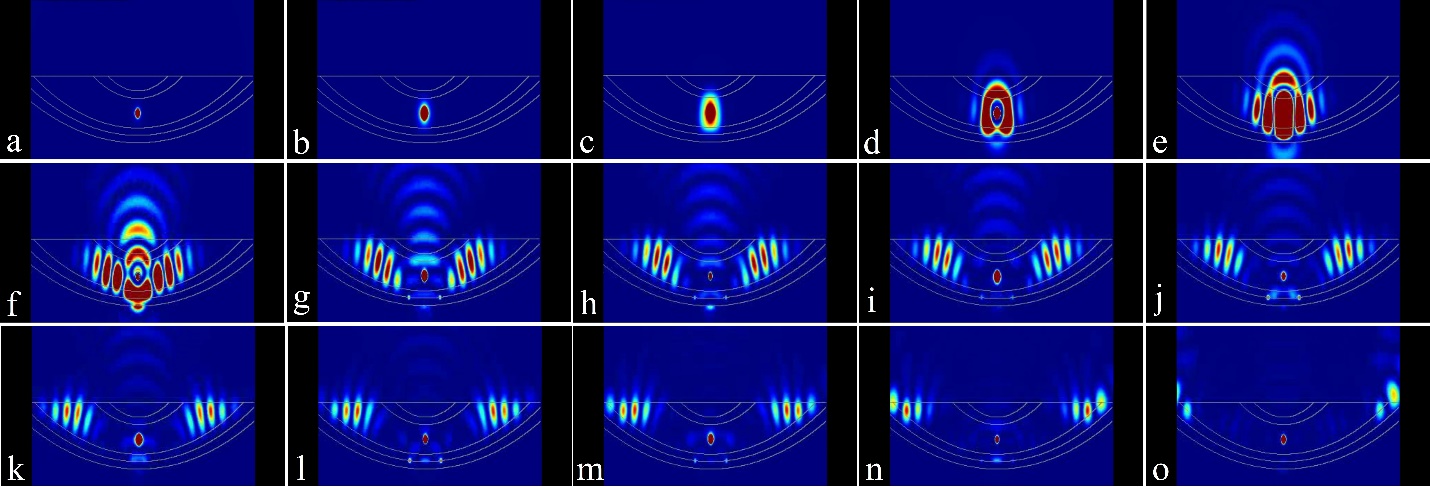


**Figure S2** shows the field profile for a 30-degree contact angle at 15 different times. Image a represents the beginning of the dipole oscillation, and subsequent images illustrate light propagation in various directions. In fig. g, the wavefront reaches the material boundary, and in Figs. h to l, we observe total internal reflection at the boundary, with the existing rays being transmitted towards the silver layer. In images m to o, we can see the wavefront interacting with the silver layer and its reflection, causing some of the light to exit at the edges. Based on these images, it can be concluded that the generated light is subjected to intense reflection in at least two regions, and travels a longer path than the channel path, leading to increased losses and decreased output light.


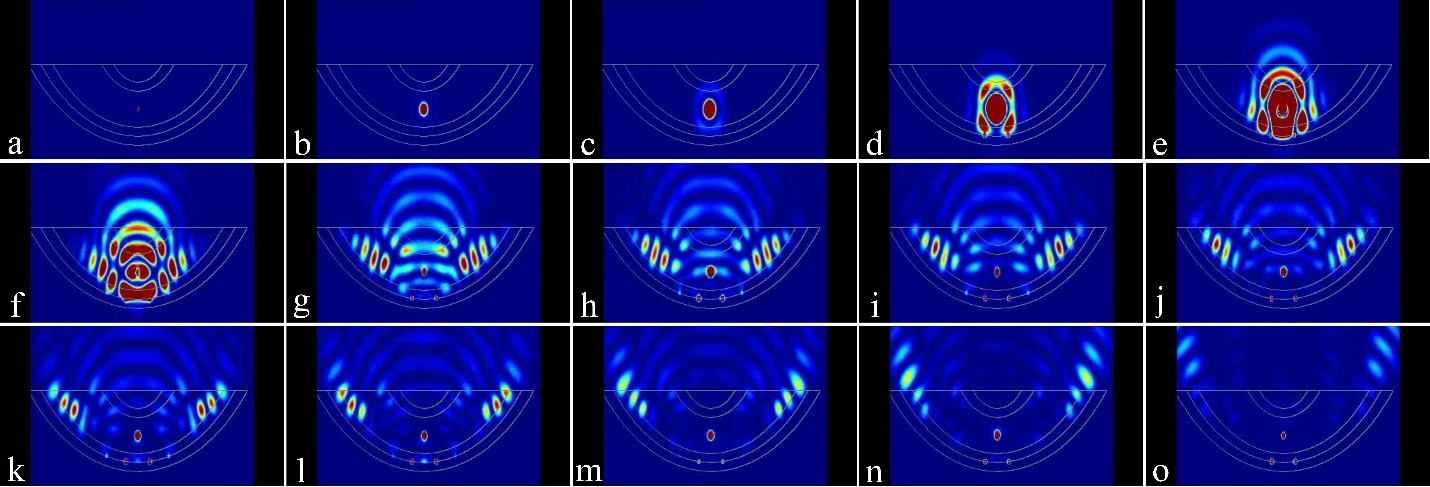


**Figure S3** The field profile for a 50-degree contact angle at 15 different times. Similar to the images in S2, the light generation and propagation process can be observed here as well. At the beginning of the wavefront collision with the boundary, seen in fig. g, the light undergoes the total reflection and is transmitted to the surroundings similar to the previous images; however, this breaking is slightly less prominent due to the increase in contact angle. In these images, output lights exit the structure with an angle from the boundary, and the output wavefronts are not perpendicular to the substrate boundary.


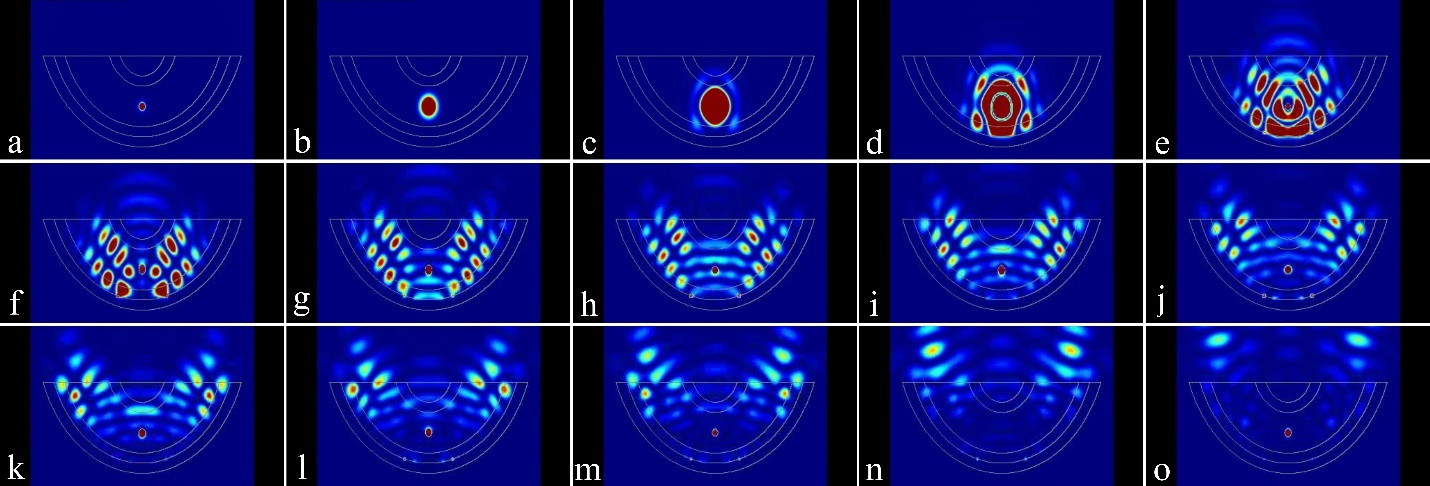


**Figure S4** The field profile for a 70-degree contact angle at 15 different times. In these images, the emitted light inside the channel collides with the substrate boundary at a nearer to perpendicular angle compared to the previous images, which can be observed from the output light.


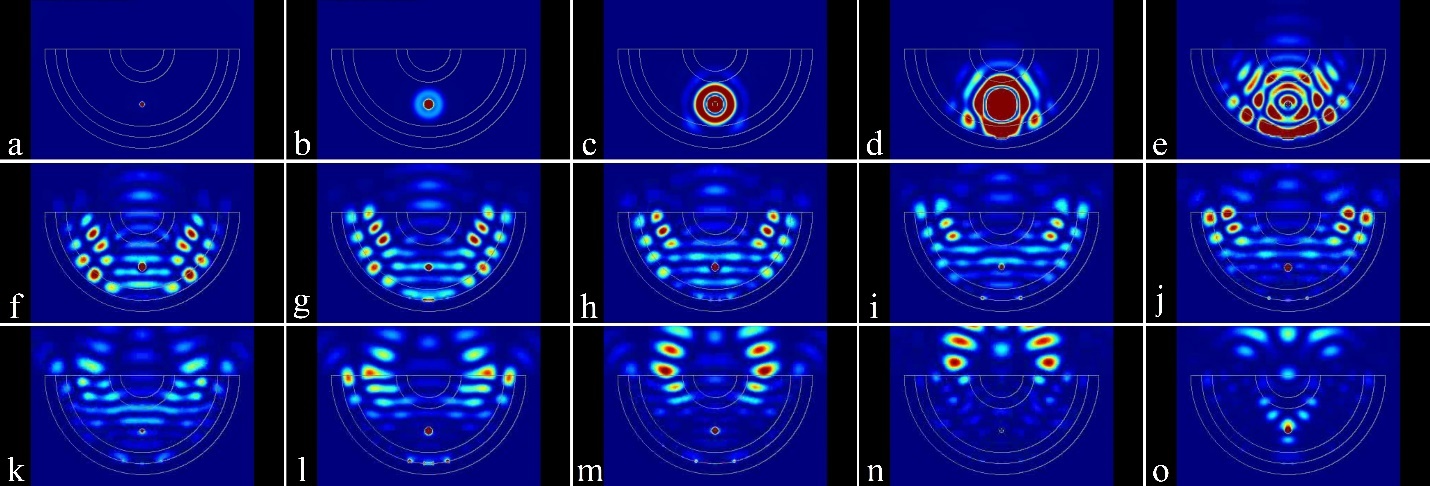


**Figure S5** The field profile for a 90-degree contact angle at 15 different times. In the above images, the wavefronts collide with the structure in such a way that the light beams hit the boundary more perpendicularly. In Fig. l to n, it can be seen that the output light is slightly inclined towards the center of the image due to slight refraction

.


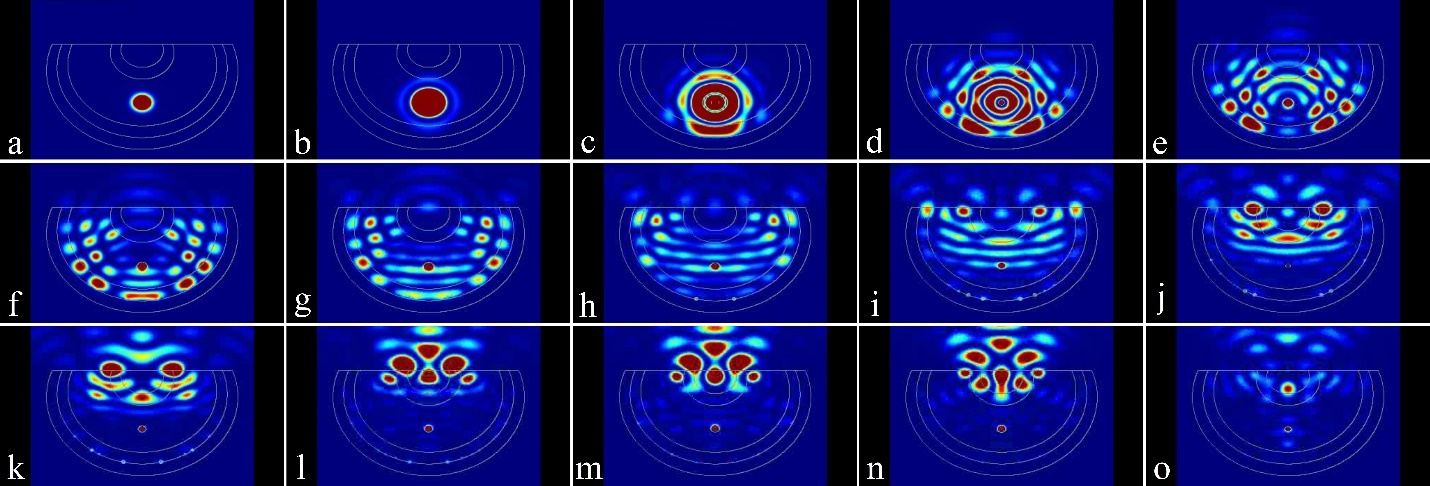


**Figure S6** shows the field profile for a contact angle of 110 degrees at 15 different times. In Fig. g, the time when the light beams hit the boundary is shown, but in Fig. i to k, it can be observed that the field intensity inside the structure has increased. This indicates that some of the incident light at the boundary has undergone the total internal reflection and returned to the interior of the structure, interfering with the existing field inside, resulting in a decrease in the output power of light and the efficiency of the structure.


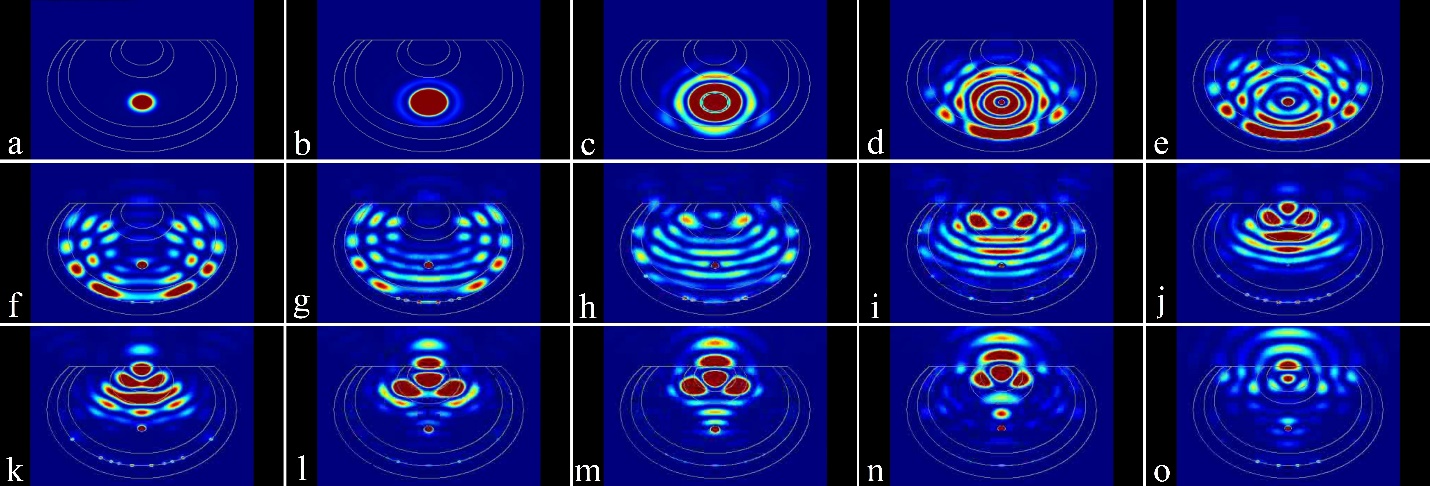


**Figure S7** shows the field profile for a contact angle of 130 degrees at 15 different times. Similar to the 110-degree case, the light beams undergo reflection and return inside. In Fig. i to n, it is clearly visible that the light is trapped inside the structure and does not exit.


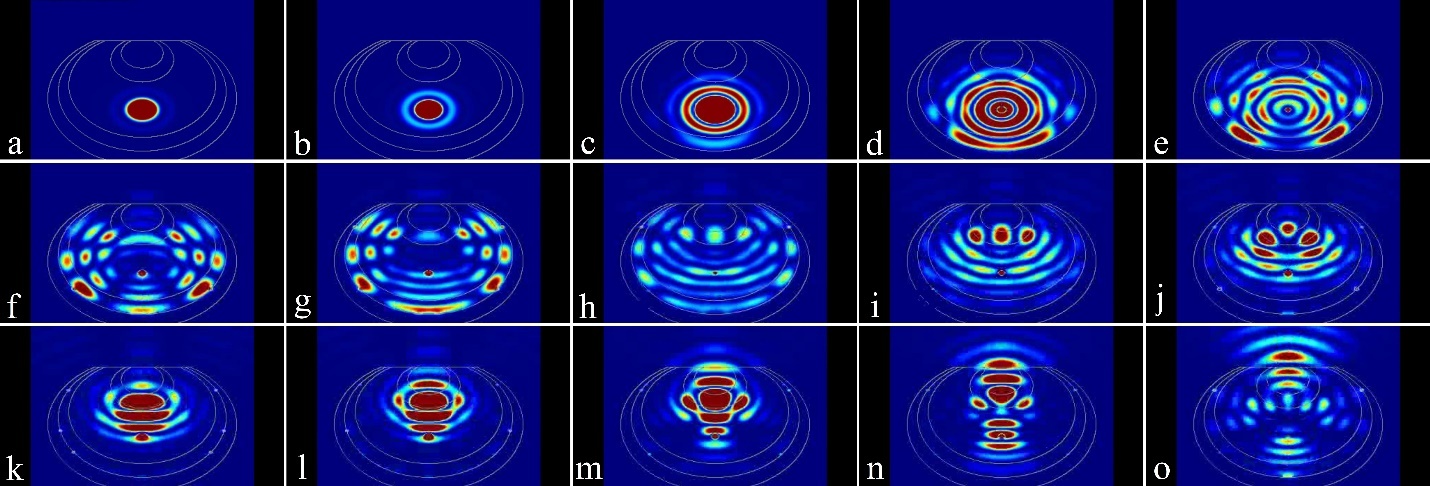


**Figure S8** shows the field profile for a contact angle of 150 degrees at 15 different times. In the above figures, it is evident that the light is completely trapped inside the structure. Due to the angle between the substrate layer boundary and perovskite channel axis, which is essentially the waveguide channel output port, light beams with angles larger than the critical angle hit the boundary and undergo the total internal reflection, getting trapped inside the structure and suffering severe losses.

**Roughness surface**

*
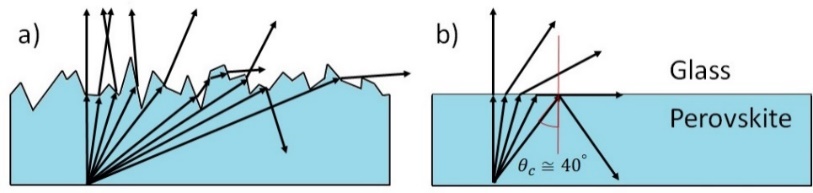
*

**Figure S9** The collision and propagation of optical rays from the perovskite layer to the glass substrate in two states: a) rough surface and b) flat surface.

**ETL & HTL thickness sweep**


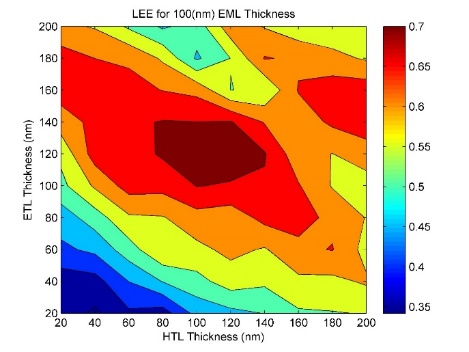

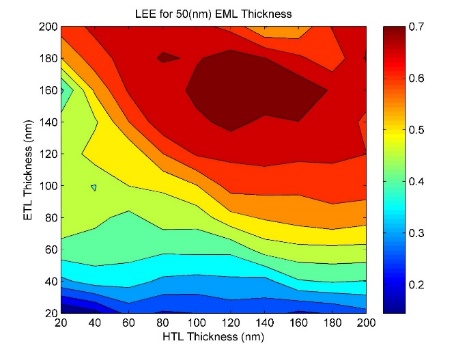


**Figure S11** The amount of light exiting the dome-shaped structure for 100 nm perovskite.

**Figure S10** The amount of light exiting the dome-shaped structure for 50 nm perovskite.


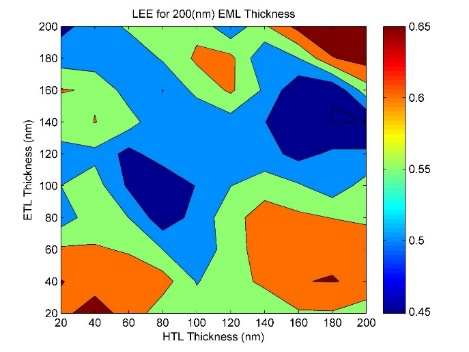

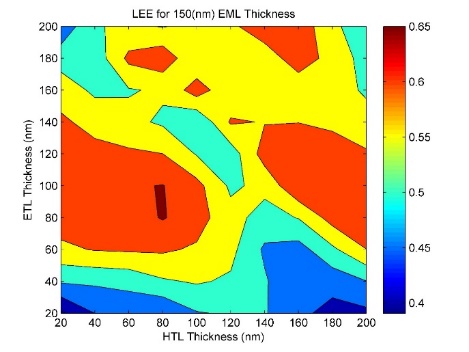


**Figure S13** The amount of light exiting the dome-shaped structure for 200 nm perovskite.

**Figure S12** The amount of light exiting the dome-shaped structure for 150 nm perovskite.


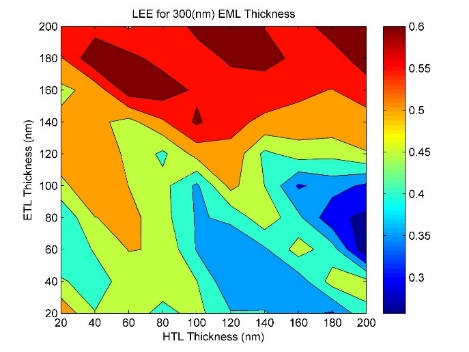

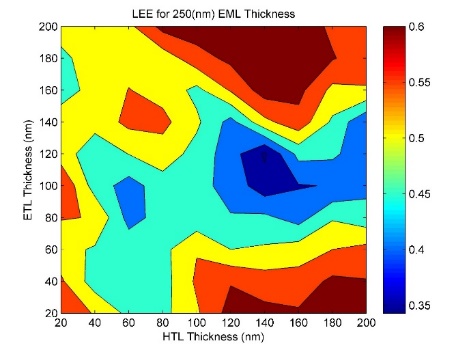


**Figure S14** The amount of light exiting the dome-shaped structure for 250 nm perovskite.

**Figure S15** The amount of light exiting the dome-shaped structure for 300 nm perovskite.


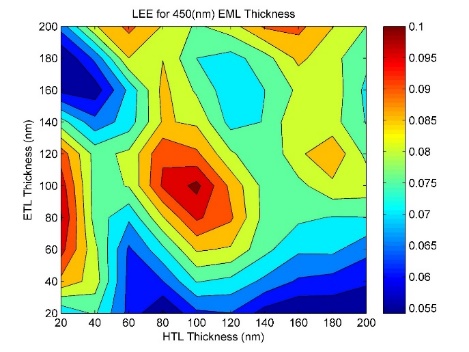

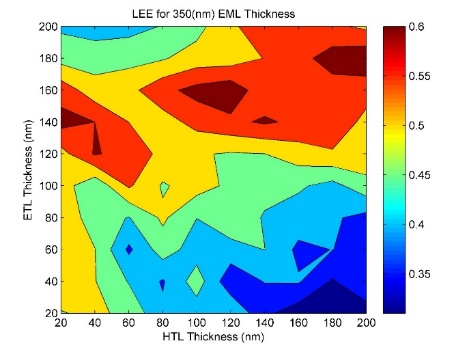


**Figure S17** The amount of light exiting the dome-shaped structure, for 450 nm perovskite.

**Figure S16** The amount of light exiting the dome-shaped structure, for 350 nm perovskite.


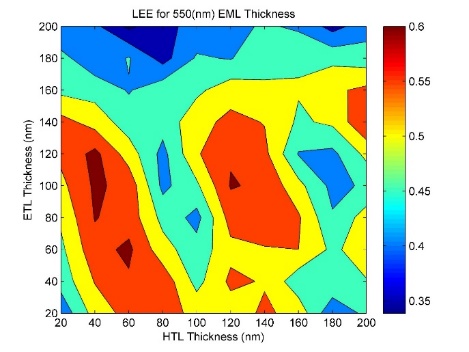

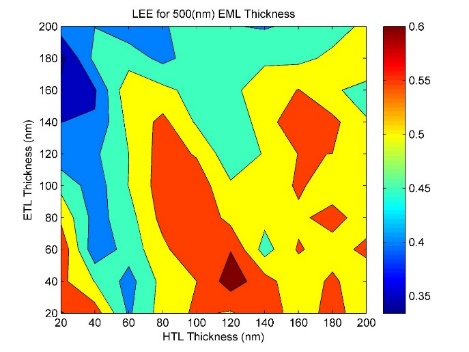


**Figure S18** The amount of light exiting the dome-shaped structure for 500 nm perovskite.

**Figure S19** The amount of light exiting the dome-shaped structure for 550 nm perovskite.


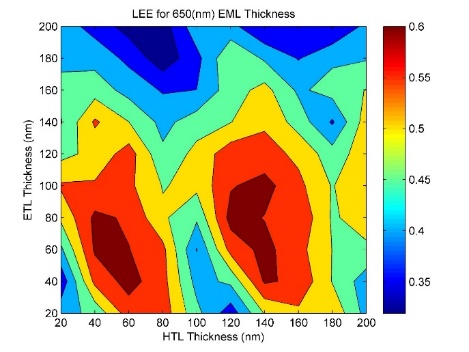

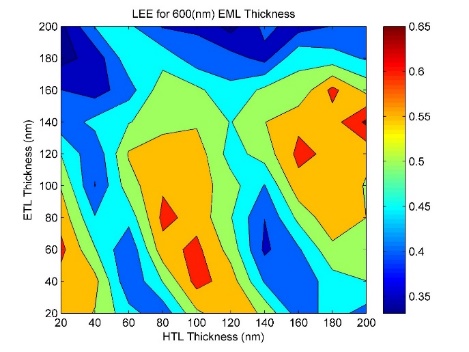


**Figure S20** The amount of light exiting the dome-shaped structure for 600 nm perovskite.

**Figure S21** The amount of light exiting the dome-shaped structure for 650 nm perovskite.


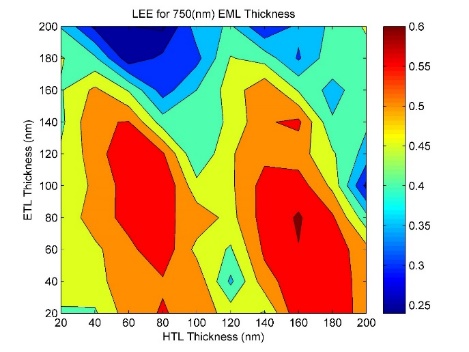

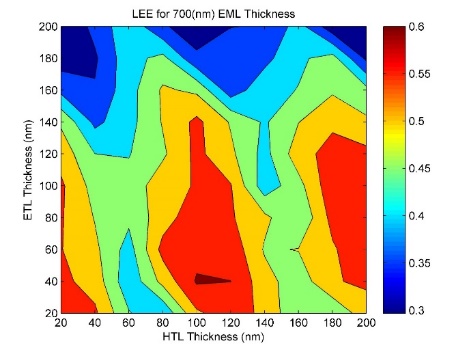


**Figure S22** The amount of light exiting the dome-shaped structure for 700 nm perovskite.

**Figure S23** The amount of light exiting the dome-shaped structure for 750 nm perovskite.


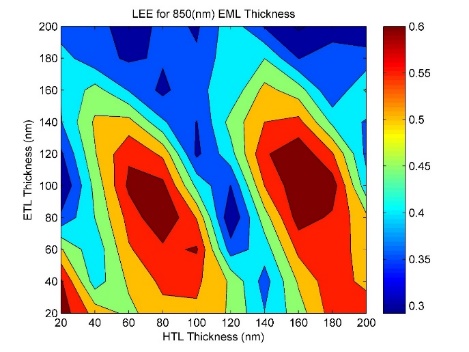

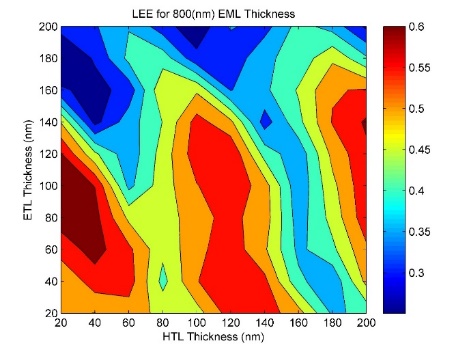


**Figure S25** The amount of light exiting the dome-shaped structure for 850 nm perovskite.

**Figure S24** The amount of light exiting the dome-shaped structure for 800 nm perovskite.


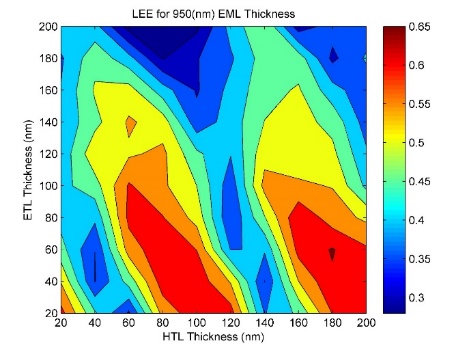

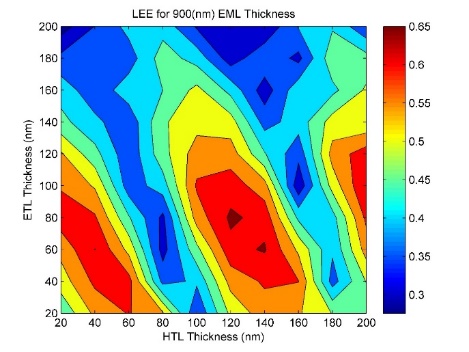


**Figure S27** The amount of light exiting the dome-shaped structure for 950 nm perovskite.

**Figure S26** The amount of light exiting the dome-shaped structure for 900 nm perovskite.

**Table S4** Max LEE (%) for Different Thicknesses of EML, ETL & HTL

| **HTL Thickness (nm)** | **ETL Thickness (nm)** | **Max LEE (%)** | **EML Thickness (nm)** |
| --- | --- | --- | --- |
| 140 | 160 | 73.77 | **50** |
| 120 | 120 | 72.32 | **100** |
| 80 | 80 | 65.38 | **150** |
| 180 | 200 | 69.59 | **200** |
| 140 | 200 | 63.56 | **250** |
| 200 | 200 | 64.45 | **300** |
| 200 | 180 | 63.71 | **350** |
| 90 | 115 | 61.26 | **400** |
| 80 | 40 | 60.18 | **450** |
| 120 | 40 | 62.57 | **500** |
| 40 | 120 | 61.41 | **550** |
| 100 | 40 | 61.93 | **600** |
| 120 | 80 | 61.89 | **650** |
| 100 | 40 | 60.56 | **700** |
| 160 | 80 | 60.5 | **750** |
| 20 | 100 | 62.34 | **800** |
| 80 | 80 | 64.83 | **850** |
| 120 | 80 | 65.93 | **900** |
| 180 | 60 | 65.36 | **950** |

**Suggested fabrication method**

The method presented in this article is merely a proposal for construction, and it is hoped that it can assist researchers in producing this structure. The construction method of this type of structure is generally divided into three stages, each of which is explained below.

The first stage involves establishing a connection between ITO domes and ultimately connecting the anode contact. Initially, a SiO2 layer is used as the substrate, and to establish a connection with the ITO domes, we need to create grooves inside this SiO2 layer. For this purpose, a photoresist layer is placed on the SiO2, and using a mask and photolithography method, we will create grooves in the photoresist material. Next, we will apply this pattern to the SiO2 using the vertical RIE etching method. Photoresist layer is formed using the spin coating process, and its material can be SU-8, which is readily available and widely used. The width of the created grooves can range from 50 to 150 nanometers to minimize significant changes in light transmission through the substrate layer. The depth of these grooves can also range from 100 to 150 nanometers. The RIE process can create a suitable and expected groove with the following conditions. etched with the gas flow rate of CHF3/Ar 10/15 sccm, RF power of 20 W, ICP power of 600 W, chamber pressure of 3 mTorr, 5 Torr of He pressure (backside cooling) and etching time of 5 minutes^1^. All stages of creating a trench in SiO2 are shown in Fig. S28.


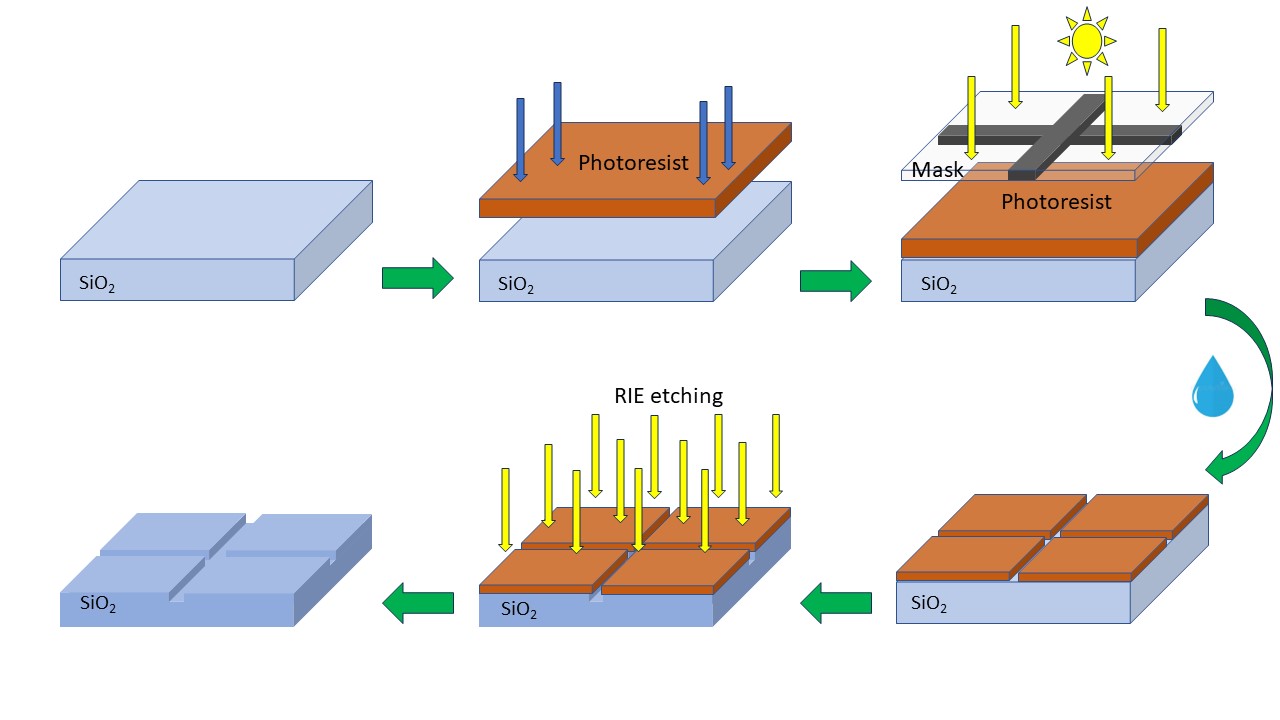


**Figure S28** The steps for creating a trench in SiO2 to establish a connection between the anode contact and ITO domes.

Then, we will deposit the ITO material on the glass using the Sputtering method. In this stage, the ITO material will cover the entire groove and the surface of the glass. Subsequently, using the photolithography method and a mask, we will create a cylindrical pattern on the photoresist material. Then, by performing vertical etching on the ITO, we will also create a cylindrical structure on the ITO, and this etching will be carried out to an extent that does not damage the ITO present in the grooves. the ITO layer of 100 to 200 nm thickness can deposit using DC magnetron sputtering system with 300 W DC power. Also, HI acid is used to ITO vertical etching and create a cylinder, and by controlling the etching time, all the ITO layer placed on the SiO2 will be etched and only ITO will remain inside the grooves^2^. The steps mentioned above are shown in Fig. S29.


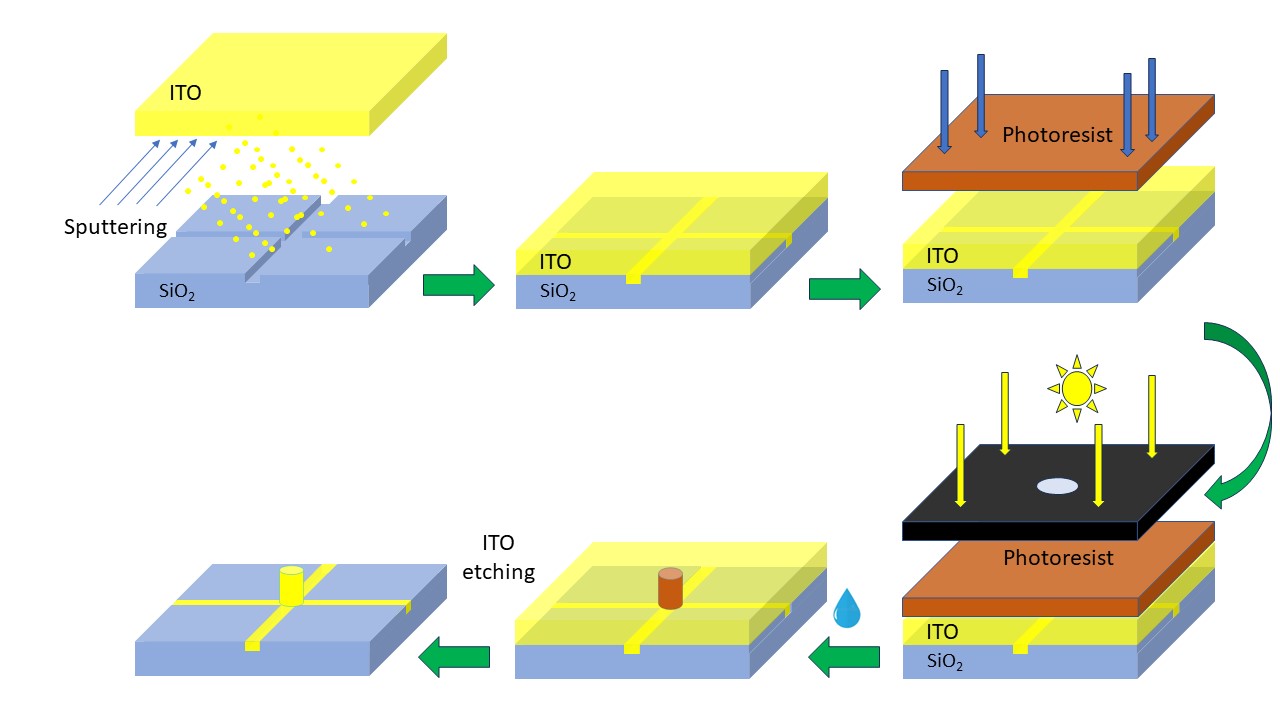


**Figure S29** The steps for creating connections between ITO domes and the anode contact using ITO.

After that, to prevent electrical connection between the ITO grooves and other materials, an insulating material such as resin or SiO2 must be applied to the structure. For the resin layer deposition, the Spin Coating method can be used, and for SiO2 deposition, the Low Temp PECVD method can be used to avoid damaging the underlying ITO layer^3-5^. Up to this stage, the connections related to the ITO have been established. Today's methods for Low Temp PECVD have grown to such an extent that it can perform this process at temperatures close to 80 degrees Celsius. The deposition of the mentioned layers can be seen in Fig. S30.


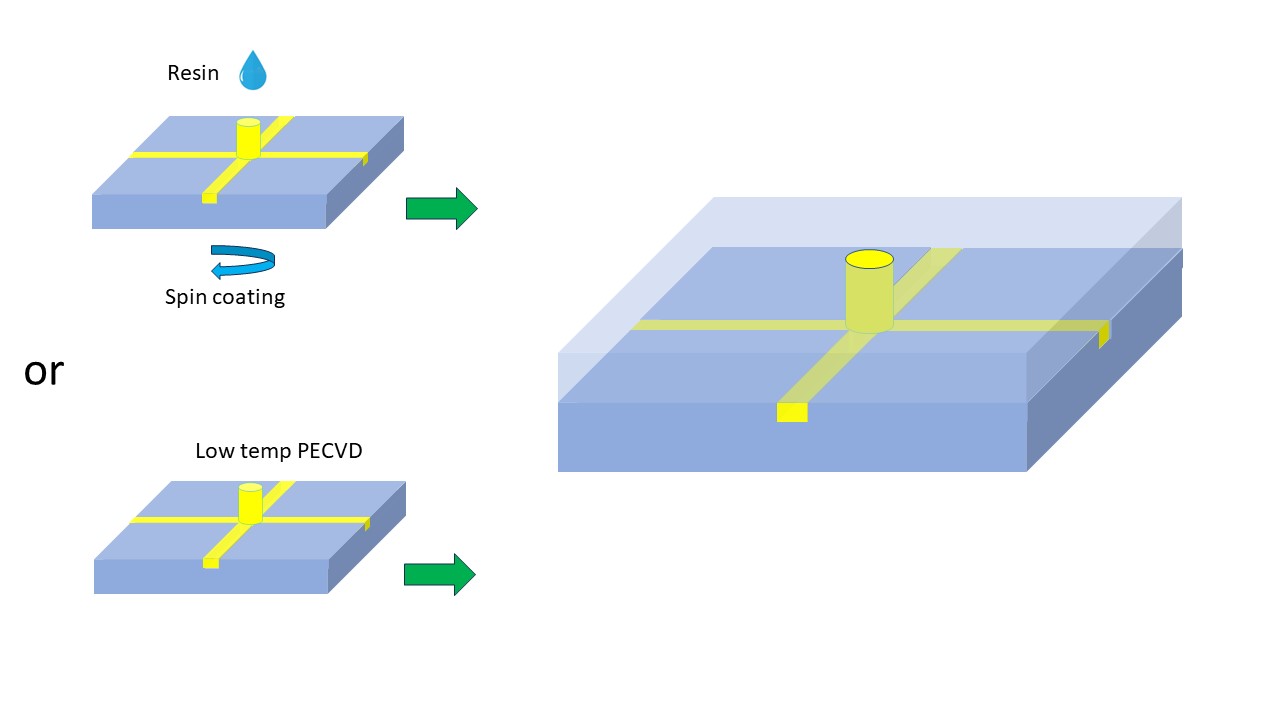


**Figure S30** Deposition an insulating layer on ITO communication paths using glass or resin.

The second stage involves the construction of ITO domes. For this purpose, first, a layer of PMMA material is applied to the structure, and then a photoresist layer is placed on it. Subsequently, a hole will be created inside the photoresist using photolithography. Next, using the RIE method, this hole will also be applied to the PMMA layer. The crucial point regarding the creation of these holes is that they must be precisely positioned on the cylinders created in the previous stages so that the ITO domes can establish electrical connections with the ITO grooves and the anode. After creating the hole, the ITO layer will be deposited on the structure using the Sputtering method. In this situation, an ITO dome will be formed inside the hole, and on the structure, there will be an ITO layer. By using the Liftoff method, the upper layers of ITO, PMMA, and photoresist will be removed, leaving only the ITO dome intact.^2,6^ PMMA layer of 250 nm thickness is spin coat over the SiO_2_ substrate at 1000 rpm for 60 s^2^. The process of RIE and sputtering is similar to the method mentioned in the previous paragraph.


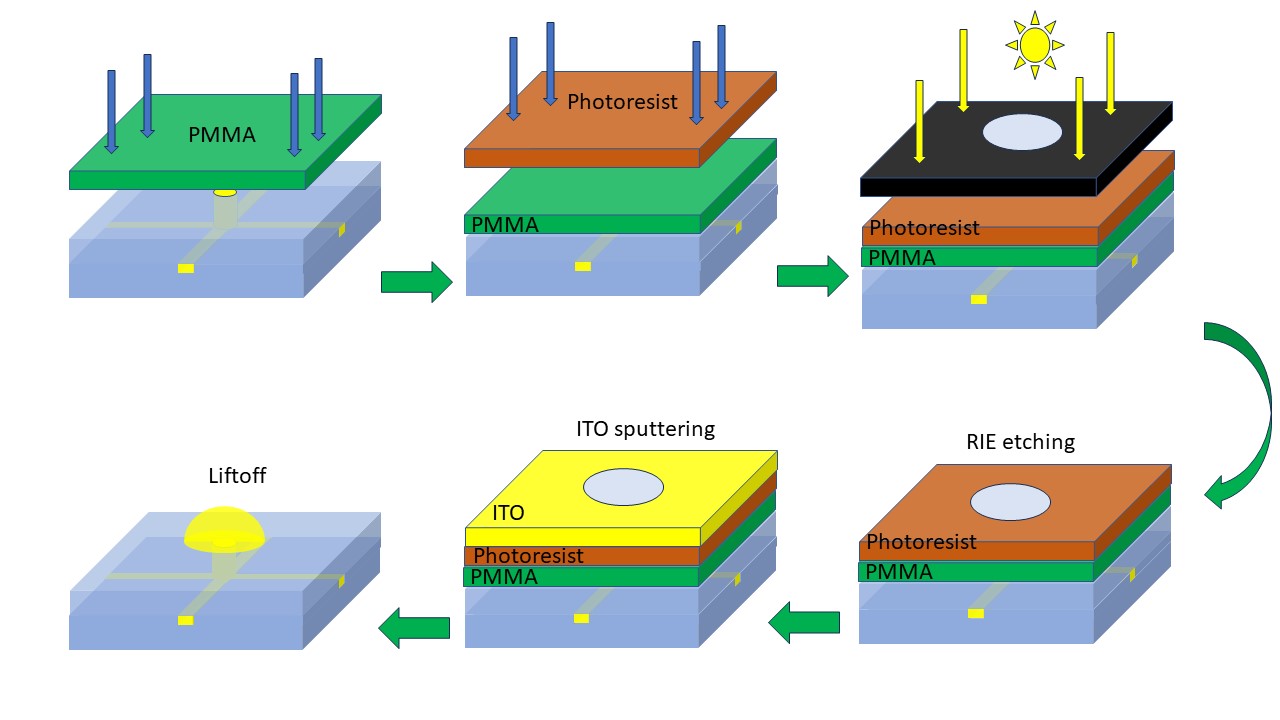


**Figure S31** ITO domes manufacturing.

The third stage of construction involves depositing the layers of PEDOT:PSS, perovskite, and F8 on the ITO domes, with the goal of maintaining their dome-shaped structure and preventing electrical connections with neighboring cells. This can be accomplished using the electrohydrodynamic (EHD) printing method, where there is an electric potential difference between the nozzle of the material and the structure's surface. This potential difference can reduce the dimensions of the droplets exiting the nozzle and increase precision in the process^7,8^. The majority of the structure's surface is covered by an insulating layer, and ITO domes are present only in a small section of the surface. The only part of the surface capable of applying voltage and carrying charge is these ITO domes. By applying a potential difference between the anode and the PEDOT:PSS nozzle, small droplets of this material can be extruded from the nozzle. The PEDOT:PSS material exiting the nozzle carries a negative electrical charge, while the ITO domes carry a positive electrical charge. This generates a strong attraction between the two materials, ensuring that the PEDOT:PSS material adheres to the ITO domes. The materials used in this method are in liquid or solution form, and it is important to ensure that they maintain their dome-shaped structure until they solidify. To achieve this, an electrostatic charge can be applied to the anode, which will make the entire ITO dome and the layer of PEDOT:PSS placed on it electrically charged. This static electric charge creates a repulsive force between the PEDOT:PSS particles, ensuring that the PEDOT:PSS coating is uniform and maintains its spherical shape. After the PEDOT:PSS has solidified, a suitable dome-shaped layer will be created on the ITO.

You can also apply the same process for the next two layers, perovskite and F8. Finally, by using Sputtering, you can deposit silver to create the cathode layer.

MAPbBr3 synthesis can perform with following method: Firstly, 0.421 mmol MABr and 0.141 mmol PbBr2 are simultaneously dissolved in dimethylformamide DMF (2ml) forming precursor solution (wt ~5%). Then the precursor solution (200μl) is printed with EHD printer. At last, the samples are settled on the thermal platform (~80 oC), and is performed thermal annealing for 20min. All the experimental procedures are must performed in a nitrogen filled glove box^9^. All of these construction stages are illustrated in Fig. S32.


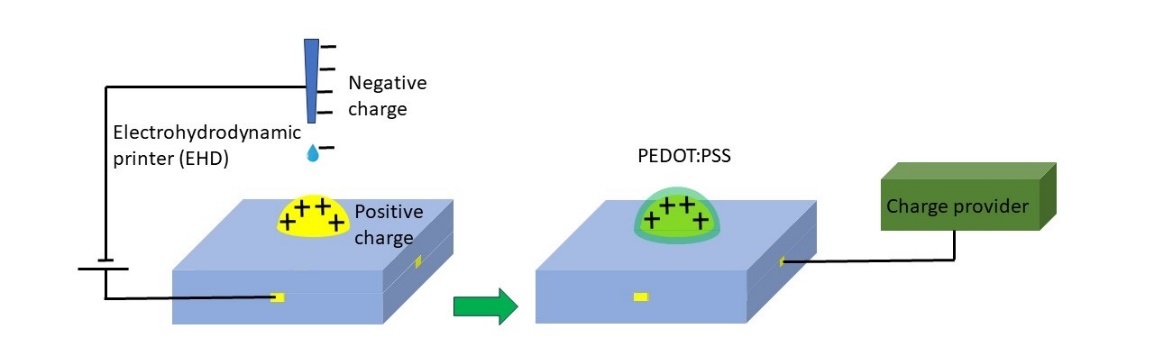


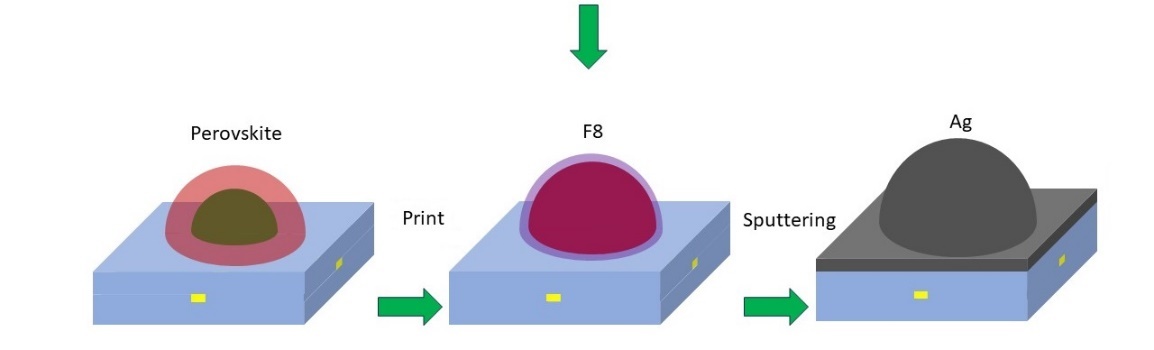


**Figure S32** Depositing different materials (PEDOT:PSS, Perovskite, F8) in a dome shape, with the help of an electrohydrodynamic printer and applying a static electric charge and Ag sputtering on the structure.

The image of the final structure in both 3D and 2D modes, along with the voltage source connection, is shown in Fig. S33.


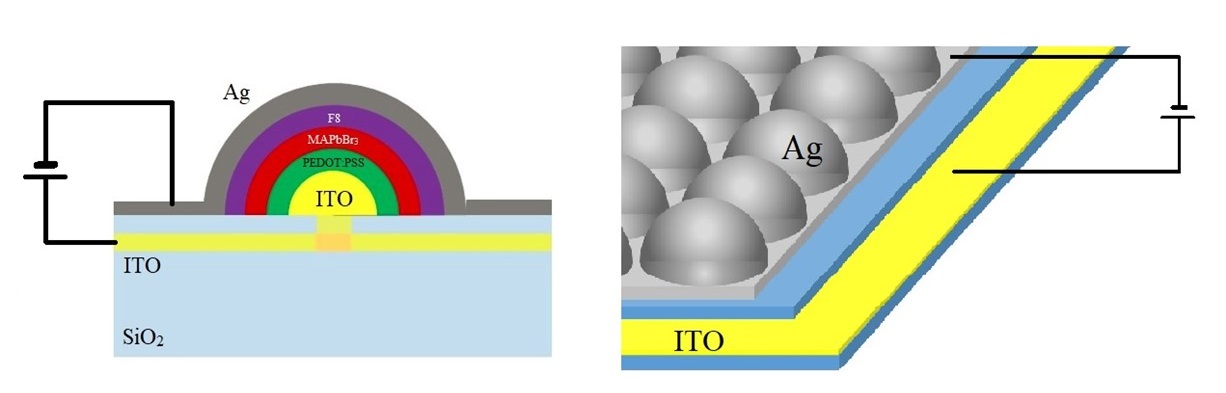


**Figure S33** 2D and 3D modes image of the final structure.

**References**

1 Alam, A. *Etching process development of SiO2 etching using inductively coupled plasma*, Itä-Suomen yliopisto, (2015).

2 Yun, J.-H., Kim, H.-S., Kumar, M. D., Park, H.-H. & Kim, J. Si photodetectors imprinted with ITO nanodomes for enhanced photodetection at NIR wavelengths. *Materials Science in Semiconductor Processing* **40**, 397-401 (2015).

3 Hirata, A., Fukasawa, M., Kugimiya, K., Karahashi, K., Hamaguchi, S. & Nagaoka, K. Damage recovery and low‐damage etching of ITO in H2/CO plasma: Effects of hydrogen or oxygen. *Plasma Processes and Polymers* **16**, 1900029 (2019).

4 Joo, S. Y. *et al.* ITO/SiO2/ITO structure on a sapphire substrate using the oxidation of ultra-thin si films as an insulating layer for one-glass-solution capacitive touch-screen panels. *Coatings* **10**, 134 (2020).

5 Son, P. K., Choi, S.-W., Kim, S. S. & Ko, S.-C. Plastic Liquid Crystal Display with Polarizers Integrated Inorganic Conducting and Alignment Layers. *Molecular Crystals and Liquid Crystals* **583**, 52-59 (2013).

6 Kumar, N., Nguyen, T. T., Park, H.-H., Lee, K., Kim, S. M. & Kim, J. Role of substrate architecture and modelling on photocurrent and photovoltage in TiO2/NiO transparent photovoltaic. *Materials Research Bulletin* **142**, 111421 (2021).

7 Mu, L. & Peng, J. in *SID Symposium Digest of Technical Papers.* 239-241 (Wiley Online Library).

8 Wang, H. *et al.* High-efficiency and high-resolution patterned quantum dot light emitting diodes by electrohydrodynamic printing. *Nanoscale Advances* **5**, 1183-1189 (2023).

9 Zhang, Z.-Y. *et al.* The role of trap-assisted recombination in luminescent properties of organometal halide CH3NH3PbBr3 perovskite films and quantum dots. *Scientific reports* **6**, 27286 (2016).
